# Mediating effects of student-perceived instructional practices in the digital gaming—reading performance relationship: a multilevel analysis of PISA 2018

**DOI:** 10.3389/fpsyg.2026.1766681

**Published:** 2026-04-10

**Authors:** Talha Göktentürk, İlayda Şeyma Oluşan

**Affiliations:** 1 Department of Turkish Education, Faculty of Education, Yildiz Technical University, Istanbul, Türkiye; 2 Department of Psychological Consultation and Guidance, Faculty of Education, Yildiz Technical University, Istanbul, Türkiye

**Keywords:** digital gaming, multilevel structural equation modeling, PISA 2018, reading performance, self-determination theory, student-perceived instructional practices

## Abstract

**Introduction:**

Digital gaming is a prevalent activity among youth, yet its impact on academic outcomes, particularly reading achievement, remains unclear. This study explores how digital gaming behaviors influence reading achievement, focusing on the mediating role of student-perceived instructional practices. Specifically, it examines the contributions of *teacher feedback*, *teacher stimulation for reading engagement*, and *teacher support* as distinct forms of instructional practice, with teacher stimulation conceptualized as an autonomy-supportive instructional practice based on self-determination theory.

**Methods:**

Using data from 606,627 15-year-old students across 79 countries in PISA 2018, a multilevel structural equation model was employed, incorporating student-level (*playing digital games*) and school-level (*teacher feedback*, *teacher stimulation*, and *teacher support*) variables. Measurement invariance was evaluated across country, world cultural region, and teacher training standards classifications to examine the cross-group comparability of the instructional-practice measures.

**Results:**

Results indicate that digital gaming is negatively associated with reading achievement. However, teacher stimulation for reading engagement shows a positive mediating effect, suggesting that autonomy-supportive instructional practices may mitigate the negative association between digital gaming and reading performance. In contrast, teacher feedback and teacher support exhibit weak or negative mediation effects, suggesting that more traditional instructional practices may be associated with less favorable indirect pathways in relation to digital gaming and reading achievement.

**Discussion:**

Overall, the findings demonstrate that instructional practices differ in their motivational significance and highlight the role of autonomy-supportive stimulation in shaping reading outcomes in contexts where digital gaming is a common out-of-school activity.

## 1 Introduction

Video games have become a dominant form of entertainment, especially among young people (Crawford and Brock, 2024). This global embrace underscores the importance of examining its effects on educational outcomes (Yu et al., 2021). However, the impact of playing digital games on academic achievement is complex to assess worldwide due to diverse social and cultural factors (Faust et al., 2020). Exploring how extracurricular gaming relates to academic success—especially in language skills—reveals broader educational implications (Cole et al., 2024). Among all language skills, reading holds a unique position in gameplay, as players frequently interact with in-game texts such as narratives, dialogues, and instructions (Rasmusson and Åberg-Bengtsson, 2015).

The nexus between digital gaming and reading achievement is inherently multifaceted. Primarily, the time dedicated to digital games may directly restrict the duration available for reading endeavors, consequently influencing reading outcomes (Drummond and Sauer, 2020). Secondly, the very substance of digital games bears indirect consequences on reading achievement. Games with an educational slant towards literacy, such as Reader Rabbit (Troy, 1994), Bookworm Adventures (Davis and Jackson, 2013), and Epistory – Typing Chronicles (Kunze, 2016), are designed to enhance reading comprehension, vocabulary acquisition, and language processing skills through interactive storytelling and text-based challenges (Ebadi and Ahmadi, 2024). In contrast, visually or aurally focused games prioritize non-textual interaction, limiting reading exposure (Schrader and Bastiaens, 2012). Despite various theoretical conjectures concerning this relationship, empirical research has been limited, particularly studies that offer multinational perspectives.

The educational implications of gaming must be viewed within global cultural and instructional diversity (Schwartz, 2009; OECD, 2021). Educational systems differ in their curricular priorities, teacher practices, and societal attitudes toward digital leisure activities, including games (IEA, 2019; OECD, 2019b). While digital gaming remains largely outside formal instruction, educators may use game-based strategies to enhance literacy (Hong et al., 2024; Magpusao, 2024). These context-specific practices underscore the need for a global perspective that captures common patterns while accounting for local variation in extracurricular gaming’s impact on reading, with a specific focus on student-perceived instructional practices. A multinational approach offers a comprehensive view of these dynamics, illuminating the role of extracurricular digital gaming across varied learning environments (Asrifan et al., 2024).

Introducing student-perceived instructional practices as mediating factors adds depth to this complex interplay by clarifying how instructional climate within schools may shape the association between extracurricular digital gaming and reading achievement across educational contexts. Within the scope of self-determination theory (SDT), student-perceived instructional practices—such as stimulation for reading engagement—can be understood as features of instructional climate that are likely to support students’ autonomy needs (Ryan and Deci, 2020). Within its specific nature, *teacher stimulation for reading engagement* and the integration of educational gaming can enhance literacy by blending leisure with academic content (Lieury et al., 2016). Alongside this autonomy-supportive dimension, teacher support and teacher feedback represent additional instructional practices through which schools may differ in linking students’ out-of-school experiences with academic reading. Through their instructional practices, teachers may influence how students engage with academic activities alongside their digital gaming habits (Ronimus et al., 2019). However, it is also important to consider whether this interplay varies according to the extent to which instructional practices are autonomy supportive or more strongly oriented toward structured instructional goals. *Teacher support*, encompassing encouragement and resource provision, may create a climate that guides students’ literacy engagement in relation to gaming (Lei et al., 2018). *Teacher feedback* may similarly link gaming to skills like comprehension, helping students view games as academically meaningful (Hwang et al., 2024). However, when instructional practices focus on approaches without autonomy-supportive features that consider students’ psychological needs, teacher support and feedback may remain more constrained by the formal aims of instruction, particularly in language lessons. Guided by SDT-related need-supportive elements, such as stimulation for reading engagement, alongside instructional practices such as feedback and support, the present study examines whether these school-level conditions help explain the association between digital gaming and reading achievement (Ryan and Deci, 2020). While theoretical models provide valuable foundations, there remains a critical need for empirical validation in cross-cultural educational environments. To address this gap, this study seeks to answer the following research questions:

1. Does the frequency of extracurricular digital game-playing behavior predict 15-year-old students’ reading achievement in PISA 2018 across participating countries?
2. Do school-level patterns in student-perceived instructional practices—namely *teacher support*, *teacher feedback*, and *teacher stimulation for reading engagement*—mediate the association between students’ digital game-playing behavior and reading achievement?
3. Does the mediating role of student-perceived instructional practices differ according to whether these practices are more autonomy supportive or more strongly oriented toward structured instruction?

## 2 Literature review

### 2.1 Digital gaming and reading achievement

Digital gaming refers to interaction with electronic games across platforms like computers, consoles, and mobile devices (Dillon, 2020). Digital gaming has been linked to both positive and negative reading outcomes (Barz et al., 2024; Kuo et al., 2025). Educational games can significantly enhance students’ reading skills (Vanbecelaere et al., 2020). However, their effects vary by design and usage, with addictive patterns potentially harming academic outcomes (Borgonovi, 2016). Integrating educational digital games into instruction has shown positive effects on reading achievement (Ronimus et al., 2019; Vanbecelaere et al., 2020). Such games have been found to promote reading comprehension, vocabulary development, engagement with complex texts, and digital literacy (Yu et al., 2021; Tay et al., 2022). They may also support higher-order skills like self-regulation and problem-solving, essential for motivation and reading success (Nietfeld, 2018; Sun et al., 2022). Conversely, digital games have been linked to negative outcomes, including academic decline (Schulz Van Endert, 2021), excessive gaming (Islam et al., 2020), aggression (Elson and Ferguson, 2014), and reduced social interactions (Bhagat et al., 2020). Taken together, digital gaming may influence reading performance positively or negatively, depending on context. Therefore, our hypothesis is as follows:

*H_1_*: Playing digital games is associated with reading achievement.

### 2.2 Search for indirect/mediating effects of digital gaming on reading achievement

As key figures in the educational process, teachers receive specialized training to meet professional learning objectives (Villegas-Reimers, 2003). This training enables them to design structured classroom activities that align with pedagogical goals and support students’ engagement and learning (Saglam et al., 2023). Although digital gaming occurs outside the classroom, its academic implications may unfold within instructional environments, particularly in reading. Teachers may reinforce students’ engagement with reading or provide structure that offsets potential distractions associated with frequent gaming (Candel et al., 2024). Rather than examining classroom-based gaming activities, this study examines how out-of-school gaming relates to reading achievement through the mediation of student-perceived instructional practices. Understanding this mediating role provides insight into how instructional environments may contribute to academic outcomes in an era when digital gaming is increasingly central to students’ lives.

#### 2.2.1 The mediating role of student-perceived instructional practices: an SDT-informed perspective

Academic motivation refers to the psychological drivers—such as goals and reasons—that underpin learning, achievement, and persistence (Urdan and Bruchmann, 2018; Wentzel and Skinner, 2022). As a dynamic construct, it is shaped by external influences and, in turn, significantly affects students’ educational behaviors (Dietrich et al., 2022). Within SDT, teachers’ practices influence classroom dynamics, particularly through autonomy-supportive behaviors that foster students’ sense of volition and relevance (Lazarides et al., 2018, 2019). Specifically, SDT emphasizes that autonomy-supportive practices can enhance students’ academic engagement, whereas more structured instructional practices such as feedback and support play important—but theoretically distinct—roles in learning environments (Van Den Bergh et al., 2013; Gamlem, 2015). Accordingly, SDT is useful here for distinguishing instructional practices that more directly support students’ autonomy from those that primarily provide structure and guidance within the learning environment.

SDT offers a framework for understanding how certain instructional behaviors may facilitate or constrain students’ psychological needs. SDT holds that individuals seek growth and well-being when their needs for autonomy, competence, and relatedness are met (Ryan and Deci, 2020). In educational settings, autonomy-supportive practices—such as teacher stimulation for reading engagement—encourage student voice, meaning-making, and self-directed involvement (De Naeghel et al., 2012). In contrast, teacher feedback (Gamlem, 2015) and teacher support (Lazarides et al., 2019) represent instructional behaviors that contribute to learning through academic guidance and assistance rather than through direct psychological need support (Huang et al., 2022). When students feel autonomous, competent, and connected, they engage more deeply in learning (Ryan and Deci, 2017); however, only some instructional practices directly target these needs, while others primarily shape the structure and clarity of learning tasks. This distinction is important for understanding how different teacher practices may relate to students’ reading development alongside broader out-of-school experiences such as digital gaming.

Although research shows that instructional environments influence reading (Huang et al., 2022) and that digital gaming relates to academic outcomes (Sánchez-Mena and Martí-Parreño, 2017), no study to date has examined how student-perceived instructional practices mediate the association between out-of-school digital gaming and reading performance. This gap matters because instructional practices may interact with students’ broader behavioral contexts, shaping academic development in subtle ways (Chu et al., 2023). While digital gaming can sometimes hinder reading achievement due to excessive screen time or reduced engagement in traditional reading (Anjum et al., 2024), it may also enhance engagement and performance when learning environments provide effective instructional support (Amzalag et al., 2024). However, without empirical evidence on how instructional practices relate to gaming behaviors, it remains unclear how these dynamics unfold across educational contexts.

PISA 2018 offers a large-scale, cross-national dataset containing indicators of digital gaming frequency, reading achievement, and student-perceived instructional practices (OECD, 2018). This study focuses on three practices—*teacher feedback*, *teacher stimulation for reading engagement*, and *teacher support*—based on their theoretical relevance and their potential as mediating variables in the relationship between extracurricular gaming and reading outcomes. Because these practices reflect student-perceived features of instructional conditions that characterize schools and were modeled as covarying constructs, their mediating roles indicate how autonomy-supportive stimulation operates alongside more structurally oriented practices such as feedback and support within the same instructional environment. By examining these three practices together, the study evaluates whether different forms of instructional climate are associated differently with reading achievement in contexts where digital gaming is prevalent.

##### 2.2.1.1 Teacher stimulation for reading engagement

Teacher stimulation for reading engagement (TSRE) refers to strategies teachers use to promote students’ interest and participation in reading (Ho and Lau, 2017, 2018; OECD, 2019b). These practices create motivating classrooms that support learning and convey teachers’ enthusiasm for reading (Pianta, 2013; Iurea, 2015). By providing targeted stimuli, teachers can make reading experiences more engaging and relevant to students’ interests (Iurea, 2015; Cattoni et al., 2024).

According to SDT, TSRE includes autonomy-supportive features that promote student choice, personal relevance, and volitional participation, thereby supporting psychological needs linked to academic engagement (De Naeghel et al., 2012; Ryan and Deci, 2020). These autonomy-supportive elements make TSRE distinct from other instructional practices, positioning it as the construct in PISA most aligned with SDT principles (OECD, 2019c). Although some gaming experiences may be narrative-rich or cognitively stimulating (Liu et al., 2024; Papadakis, 2018), their connection to TSRE has not been directly studied. Prior work suggests that both reading engagement and gaming engagement can reflect students’ intrinsic interests, but excessive gaming without academic scaffolding may hinder literacy development (Anthony et al., 2021).

Given TSRE’s autonomy-supportive nature, this study investigates its role as a potential mediator in the association between extracurricular gaming and reading achievement. Within this conceptual framework, TSRE is treated as an autonomy-supportive instructional practice that may help connect students’ out-of-school experiences with reading-related engagement in school, thereby providing a theoretically grounded explanation for its mediation potential. Therefore, our hypotheses are:

*H_2a_*: Extracurricular digital game-playing behavior is associated with teacher stimulation for reading engagement.

*H_2b_*: Teacher stimulation for reading engagement is associated with reading achievement.

##### 2.2.1.2 Teacher feedback

Teacher feedback is a critical component of instructional practice, offering targeted information to support performance and learning goals (Carless and Winstone, 2023; Saglam and Goktenturk, 2024). Although feedback can influence students’ motivation, it is generally understood as a structured form of instructional guidance rather than a direct form of psychological need support. Within SDT, feedback may enhance students’ sense of competence when delivered in an informational rather than controlling manner (Deci and Ryan, 2012). However, in the context of PISA, teacher feedback is measured as performance-oriented guidance rather than autonomy- or competence-supportive feedback (OECD, 2019c).

Students’ gaming habits may relate to different patterns of academic engagement, which in turn may coincide with variation in the types of feedback students perceive in their classrooms (Ronimus et al., 2019; Facchino et al., 2025). For example, students demonstrating persistence may report receiving more positive or encouraging feedback, while students struggling with reading may report more corrective input (Liu and Israel, 2022; Zou et al., 2022). Such differentiated responses reflect teachers’ instructional sensitivity to students’ academic behaviors and can guide students in recognizing the relevance of their learning strategies (Hao et al., 2022; Grønli et al., 2025). As a form of instructional guidance, well-structured feedback may help students frame academic and extracurricular activities—including digital gaming—in more productive ways (Montgomery and Baker, 2007; Lawrence and Sherry, 2021).

While feedback is widely recognized as essential for reading improvement (Ma et al., 2023; Yang et al., 2021), its role in shaping how students balance digital gaming and reading performance has not been specifically addressed. Because PISA’s feedback construct reflects structured instructional practice rather than autonomy-supportive competence feedback, its mediating role is conceptualized within the broader instructional environment reflected in students’ shared perceptions of classroom practice. This study therefore examines whether teacher feedback is associated with extracurricular gaming and whether it contributes to reading achievement alongside other instructional practices. Accordingly, the hypotheses for this study are:

*H_3a_*: Extracurricular digital game-playing behavior is associated with teacher feedback.

*H_3b_*: Teacher feedback is associated with reading achievement.

##### 2.2.1.3 Teacher support

Teacher support includes strategies that promote academic and emotional development through a positive classroom climate (Lei et al., 2018). Although supportive interactions can foster students’ sense of relatedness in some instructional contexts (Banerjee and Halder, 2021; Sağlam et al., 2023), the PISA 2018 teacher support construct primarily reflects structured academic assistance and responsiveness to learning needs rather than direct measures of relatedness-supportive practice (OECD, 2019c). Supportive behaviors such as providing extra help, showing interest in students’ learning, and offering clarification can shape students’ perceptions of the classroom environment and encourage sustained academic engagement (Cabellos et al., 2023; Toh and Kirschner, 2023). Such guidance may help students interpret their learning behaviors more effectively and remain engaged in reading tasks (Jensen et al., 2019; Kokandy, 2021). However, the effectiveness of teacher support may depend on how well it responds to students’ individual learning characteristics and needs (Felicia, 2009). When support emphasizes curricular demands without addressing students’ diverse preferences or engagement patterns, its impact on learning may be limited (Blume, 2020).

While gaming can cause distraction or overuse (Jeong et al., 2017), it may also contribute to learning when instructional environments offer appropriate structure and support (Hong et al., 2009). Students’ extracurricular gaming habits may correspond with different patterns of academic engagement, and such engagement differences can shape how students perceive the support available in their classrooms (Usta and Şahan, 2024). Because teacher support in PISA reflects student-perceived instructional conditions within schools, its association with gaming is interpreted as part of the broader instructional context rather than as a direct teacher response to students’ gaming behaviors (OECD, 2019c). Research consistently links teacher support to reading achievement and engagement (Ma et al., 2021; Tao et al., 2022). In this study, teacher support is therefore examined as an instructional factor that may be associated with both extracurricular gaming and reading achievement within the broader classroom environment. Accordingly, the hypotheses for this study are:

*H_4a_*: Extracurricular digital game-playing behavior is associated with teacher support.

*H_4b_*: Teacher support is associated with reading achievement.

**Figure 1:**
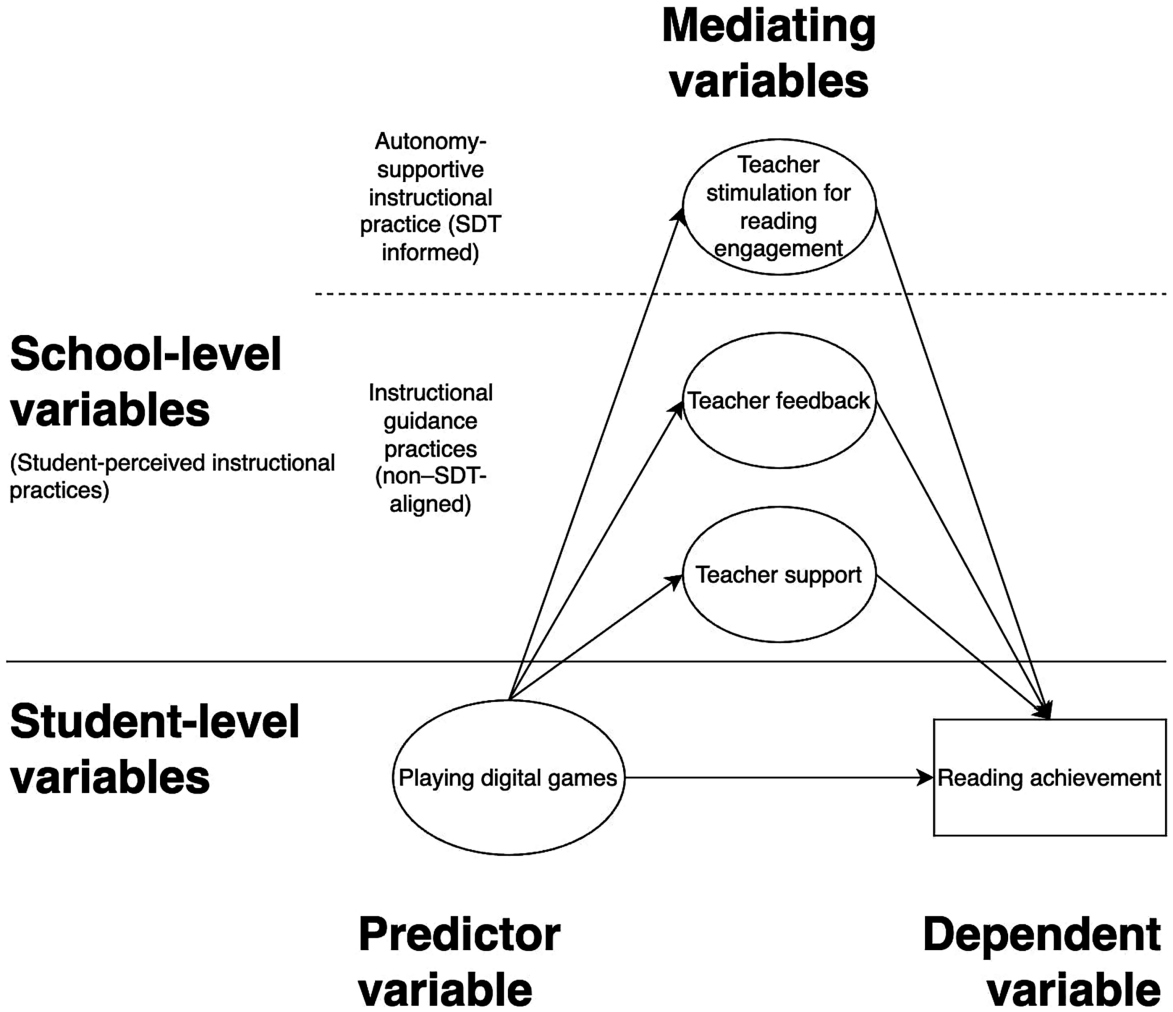
Hypothesized conceptual model.

Existing research on teacher–student interactions is often fragmented and localized, and few studies have examined the mediating role of instructional support in the association between digital gaming and reading achievement using large-scale, cross-national data. This study addresses that gap through the conceptual model in Figure 1.

### 2.3 Why PISA? Understanding digital gaming, reading, and student-perceived instructional practices

Cross-national data are needed to understand these dynamics across educational contexts (Li et al., 2025). Programme for International Student Assessment (PISA) provides an ideal platform due to its comprehensive, global scope (OECD, 2023). It assesses 15-year-olds’ skills in reading, math, and science, while also capturing digital behaviors and instructional practices (Schleicher, 2023). Unlike small-scale datasets, PISA supports global comparisons and reveals long-term trends in learning and technology use (Hu and Yu, 2021). Most PISA-based studies focus on digital reading (Luo et al., 2024), ICT environments (Acun Çelik et al., 2024), or the broader digital technology roles (Díaz et al., 2024). A few studies have linked gaming behavior to reading outcomes, with results varying by type, frequency, and context (Basaran and Simsek, 2024). Other studies have examined instructional practices—such as autonomy-supportive teaching or feedback—and their associations with student engagement and achievement (Ma et al., 2021; Suárez-Mesa and Gómez, 2024). However, no PISA-based study has examined whether different forms of instructional practice—including autonomy-supportive and non–autonomy-supportive practices—mediate the relationship between extracurricular gaming and reading achievement. This study integrates these dimensions by examining how student-perceived instructional practices, treated as contextual features of schools’ instructional environments, are associated with gaming and reading performance across diverse educational contexts.

## 3 Method

### 3.1 Data

This study utilizes data from the 2018 cycle of the Programme for International Student Assessment (PISA), conducted by the Organisation for Economic Co-operation and Development (OECD) since 2000. The 2018 assessment, administered via computer, includes measures of students’ digital game-playing behavior, reading achievement, and student-perceived instructional practices (OECD, 2019b). The dataset encompasses a total sample of 606,627 students (49.8% female) from 21,752 schools across 79 countries. In accordance with PISA’s complex sampling design, the final student sampling weight (W_FSTUWT) was used in the multilevel models, and the nested sampling structure was modeled by specifying school identifiers (CNTSCHID, combined with country where needed) as the clustering variable in the MLSEM (OECD, 2018). Before estimation, W_FSTUWT was normalized within each plausible-value analytic sample by dividing each weight by the sample mean weight and then entered as the sampling-weight variable in each model. Additional sampling information is provided in Supplementary Table 3.

### 3.2 Measures

The multilevel model includes student-level variables—playing digital games and reading achievement—and school-level student-perceived instructional practices: feedback, stimulation for reading engagement, and support. Descriptive statistics and reliability information are presented in Supplementary Tables 1, 2, 4. Variables with Cronbach’s alpha values of approximately 0.70 or higher were considered to demonstrate acceptable internal consistency.

#### 3.2.1 Student-level variables

##### 3.2.1.1 Digital gaming

Playing digital games (PDG) was measured using three items in the PISA 2018 student questionnaire, capturing engagement in (1) single-player games, (2) collaborative online games, and (3) online games via social networks. Responses were recorded on a five-point Likert scale ranging from “Never or hardly ever” (1) to “Every day” (5). The items were combined into a composite score representing overall digital gaming behavior, with acceptable internal reliability (Cronbach’s *α* = 0.73). Importantly, the PISA 2018 digital gaming measure captures the frequency of general extracurricular gaming behavior and does not distinguish game genre, educational purpose, or literacy-related design features; therefore, it cannot differentiate between educational games and entertainment-oriented games. Item details and response options are provided in Supplementary Table 2.

##### 3.2.1.2 Reading achievement (RA)

RA is one of PISA’s core assessment domains and was evaluated through computer-based adaptive testing, with item difficulty adjusted to student responses. The assessment was administered in students’ native languages. To account for measurement error, PISA reports ten plausible values per student rather than a single score. In the present study, the MLSEM was estimated separately for each plausible value (PV1READ–PV10READ), and the parameter estimates from the ten plausible-value runs were pooled following Rubin’s rules. That is, each plausible value was treated as a separate analysis, the model specification was held constant across runs, and the resulting coefficients and standard errors were combined using Rubin-style pooling procedures.

#### 3.2.2 School-level variables

##### 3.2.2.1 Teacher feedback (TF)

Teacher feedback reflects students’ perceptions of instructional guidance during language lessons. It was measured using three items: (1) feedback on students’ strengths, (2) identification of areas for improvement, and (3) suggestions for enhancing performance. Responses were recorded on a four-point Likert scale from “Never or almost never” (1) to “Every lesson or almost every lesson” (4), with higher scores indicating more frequent feedback. Internal reliability was high (Cronbach’s *α* = 0.85). Response distributions and reliability statistics appear in Supplementary Table 2.

##### 3.2.2.2 Teacher stimulation for reading engagement (TSRE)

TSRE represents an autonomy-supportive instructional practice and is the only measure conceptually aligned with SDT principles. Four items asked students how often teachers: (1) encourage them to express opinions about texts, (2) connect reading to personal experiences, (3) link content to prior knowledge, and (4) pose questions that foster engagement. Responses were on a four-point Likert scale from “Never or hardly ever” (1) to “In all lessons” (4), with higher values reflecting greater stimulation. Reliability was strong (Cronbach’s α = 0.85). Additional scale details are available in Supplementary Table 2.

##### 3.2.2.3 Teacher support (TS)

Teacher support captures students’ perceptions of academic assistance and instructional responsiveness. Four items assessed the extent to which teachers: (1) show interest in students’ learning, (2) provide additional help, (3) assist with comprehension, and (4) persist until students understand the material. Responses were collected on a four-point Likert scale from “Every lesson” (1) to “Never or hardly ever” (4) and reverse-coded so that higher values indicate greater support. Reliability was high (Cronbach’s α = 0.87). The scale details are available in Supplementary Table 2.

### 3.3 Data analysis

Before conducting analyses, missing data patterns were examined. Digital gaming variables had the highest missing rate (approximately 45%), while school-level variables ranged from 6.7 to 10%. Little’s MCAR test—along with Hawkins’ and non-parametric tests—rejected the MCAR assumption at *p* < 0.05 for both student- and school-level variables, indicating the data were not missing completely at random (Jamshidian et al., 2014). Pattern-mixture sensitivity analyses (MNAR) showed stable model fit and path estimates, supporting the plausibility of the missing-at-random (MAR) assumption (Enders, 2022). Accordingly, multiple imputation was applied, following best practices in handling MAR data (Garson, 2015).

Missing data were addressed using multiple imputation via the R package mice (Buuren and Groothuis-Oudshoorn, 2011). This method was preferred over listwise deletion due to its ability to reduce bias and maximize use of available data (Enders, 2022). Student- and school-level variables were imputed jointly using linear regression (*norm.predict*), suitable for continuous data (Henrickson et al., 2015). Key assumptions—linearity and normality—were confirmed through scatterplots and Q-Q plots, which showed expected associations and normally distributed residuals (Roback and Legler, 2021). Diagnostic visuals are provided in Supplementary Figures 1, 2. All variables with missing values, including PDG, TF, TSRE, and TS, were treated as both predictors and outcomes during imputation to preserve their multivariate relationships and minimize bias.

Before testing the mediation model, we evaluated the factorial structure of the three instructional practice variables across all participating countries to account for cultural, contextual, and teacher training differences. Three classification systems were applied. First, the country (CNT) was used to assess construct consistency across the 79 participating national samples (OECD, 2018). Second, the World Cultural Regions (WCR) index, based on Schwartz’s cultural framework (2009), was used to represent broader cultural groupings across participating countries. Third, a teacher training standards (TTS) index was created using the UNESCO ISCED-T 2021 framework to reflect minimum qualifications for upper secondary teachers across countries (UNESCO, 2022). Together, these indices supported the examination of cross-cultural validity and ensured that the instructional practice constructs were interpreted consistently across diverse national contexts.

Measurement invariance was tested separately for each of the three classification systems—CNT, WCR, and TTS—to evaluate construct consistency prior to multilevel structural equation modeling. Using multi-group confirmatory factor analysis (MGCFA) in R’s *lavaan* package, we estimated a sequence of configural, metric, and scalar invariance models for each classification system independently (Hirschfeld and von Brachel, 2014; Brown et al., 2017). For each sequence, model fit was evaluated at each level of invariance, and decisions were based on overall fit across models (CFI > 0.90, TLI > 0.90, SRMR ≤ 0.08, RMSEA < 0.08) (Clark and Bowles, 2018; Ali et al., 2026). The fit statistics for the configural, metric, and scalar models are reported in Supplementary Table 7 and support the comparability of the instructional-practice constructs across CNT, WCR, and TTS (Mackinnon et al., 2022). Therefore, partial invariance was not tested for CNT, WCR, and TTS (Van Dijk et al., 2022).

To assess whether the mediation was partial or full, the direct effect of playing digital games on reading achievement was tested before estimating the multilevel structural equation model (MLSEM) (Little et al., 2007). MLSEM was used due to the hierarchical structure of the PISA data, with students nested within schools (OECD, 2018). This approach enables simultaneous analysis of student- and school-level variables, accounting for intra-school correlations and improving estimation accuracy (Rosseel, 2012). Because PDG and RA were measured at the student level within a nested dataset, MLSEM separates their within-school and between-school components (OECD, 2018). Accordingly, the indirect pathways involving TF, TSRE, and TS were estimated at the between-school level and are interpreted as contextual pathways within schools’ instructional environments (OECD, 2019c). More specifically, the model tests whether between-school variation in digital game-playing behavior is associated with between-school variation in student-perceived instructional practices, and whether these school-level differences are, in turn, associated with reading achievement. The nested structure of students within schools was modeled directly in the MLSEM, and school identifiers (CNTSCHID, combined with country where required to ensure unique school clusters across the pooled international dataset) were specified as the clustering variable (OECD, 2018). In each model, the final student weight (W_FSTUWT) was normalized within each plausible-value analytic sample and then specified as the sampling-weight variable (OECD, 2018). The MLSEM was estimated separately for each of the ten reading plausible values (PV1READ–PV10READ), while keeping the model specification identical across runs. The resulting PV-specific parameter estimates, including standardized coefficients, were then pooled according to Rubin’s rules (Rubin, 2018; Aparicio et al., 2021). Thus, the analysis followed a repeated-estimation approach across plausible values rather than a single-score analysis of reading achievement (OECD, 2018). To support replicability, sample syntax for the multilevel analysis is provided in Supplementary Table 5. As an additional robustness check, the final two-level model was re-estimated with gender included as an additional covariate, and the resulting direct and indirect estimates are reported in Supplementary Table 8 (González-González et al., 2022). Robust maximum likelihood estimation was used throughout, and indirect effects were computed within each plausible-value run and then pooled across runs (Beauducel and Herzberg, 2006; Tibbe and Montoya, 2022). Effect significance was determined by the confidence interval method (Zhao et al., 2010). Model fit was evaluated using the Comparative Fit Index (CFI), Tucker-Lewis Index (TLI), and Root Mean Square Error of Approximation (RMSEA). The CMIN/df was excluded due to its sensitivity to large sample sizes, which can distort results (Barrett, 2007). CFI and TLI values above 0.90 indicated acceptable fit, while values ≥ 0.95 reflected excellent fit (Hu and Bentler, 1999; Kline, 2005). RMSEA values below 0.08 were interpreted as acceptable fit (Xia and Yang, 2019).

## 4 Results

### 4.1 Preliminary analysis

Multi-group confirmatory factor analysis (MGCFA) indicated that the instructional-practice constructs were comparable across the classification systems and were interpreted consistently across the 79 country subgroups. Measurement invariance was established at the configural, metric, and scalar levels, indicating structural, loading, and intercept equivalence, respectively (Mackinnon et al., 2022). This process is critical in cross-cultural research to ensure that latent constructs—such as teacher feedback, stimulation, and support—are comparable across cultural contexts (Jeong and Lee, 2019). Fit indices supported invariance across all three levels (CFI > 0.90, SRMR ≤ 0.08, RMSEA < 0.08). The fit statistics for the configural, metric, and scalar models are reported in Supplementary Table 7. Overall, the invariance findings support the comparability of factor structure, factor loadings, and item intercepts across all three classification systems (i.e., TTS, WCR, and CNT).

Variance decomposition showed that approximately 35.9% of the variance in reading achievement occurred at the school level, while 64.1% was attributable to student-level differences. The intraclass correlation coefficient (mean ICC across plausible values = 0.359) therefore indicates substantial between-school variation, supporting the use of multilevel modeling to account for the dependence of students nested within schools (Kong et al., 2022; Baysu et al., 2023). Given this magnitude of clustering, multilevel modeling is justified, as it more accurately partitions variance and accounts for dependence among students within schools (Dong et al., 2016; OECD, 2018).

Table 1 presents the factor-level descriptive statistics and latent correlations among the mediating variables. Item-level descriptive statistics, including means and standard deviations for individual items, are available in Supplementary Table 1. All latent correlations were moderate, positive, and statistically significant (*p* < 0.001), ranging from 0.37 to 0.49. The strongest correlation was between teacher feedback and teacher stimulation for reading engagement (*r* = 0.49), suggesting a tendency for these practices to co-occur. Teacher support was also positively associated with both feedback (*r* = 0.37) and stimulation (*r* = 0.41), reflecting the interconnected nature of instructional climates at the school level.

**Table 1:** Descriptive statistics and correlation matrix of mediating variables.

Category	Variable	Mean	Std. deviation	Range	TF	TSRE	TS
Student-level							
Independent variable	PDG	2.50	0.89	1–5			
School-level							
Mediating variables	TF	2.38	0.81	1–4	1		
	TSRE	2.65	0.76	1–4	0.49***	1	
	TS (reverse coded)	3.18	0.76	1–4	0.37***	0.41***	1

PDG, playing digital games; TF, teacher feedback; TSRE, teacher stimulation for reading engagement; TS, teacher support. ***p < 0.001.

Descriptive statistics revealed variation in student-perceived instructional practices. Teacher support had the highest mean (*M* = 3.18, *SD* = 0.76), suggesting it is perceived as the most prevalent, while teacher feedback had the lowest (*M* = 2.38, *SD* = 0.81), indicating it may occur less frequently. Teacher stimulation for reading engagement was intermediate (*M* = 2.65, *SD* = 0.76), with variability across classrooms. The direct effect of PDG on reading achievement was significant and negative (*β* = −0.21, *p* < 0.001, 95% CI [−0.232, −0.196]), indicating that higher gaming frequency is associated with lower reading performance. With measurement validity and variance components established, the analysis proceeded to test indirect associations using multilevel structural equation modeling (MLSEM).

### 4.2 Multilevel structural equation modeling

**Figure 2:**
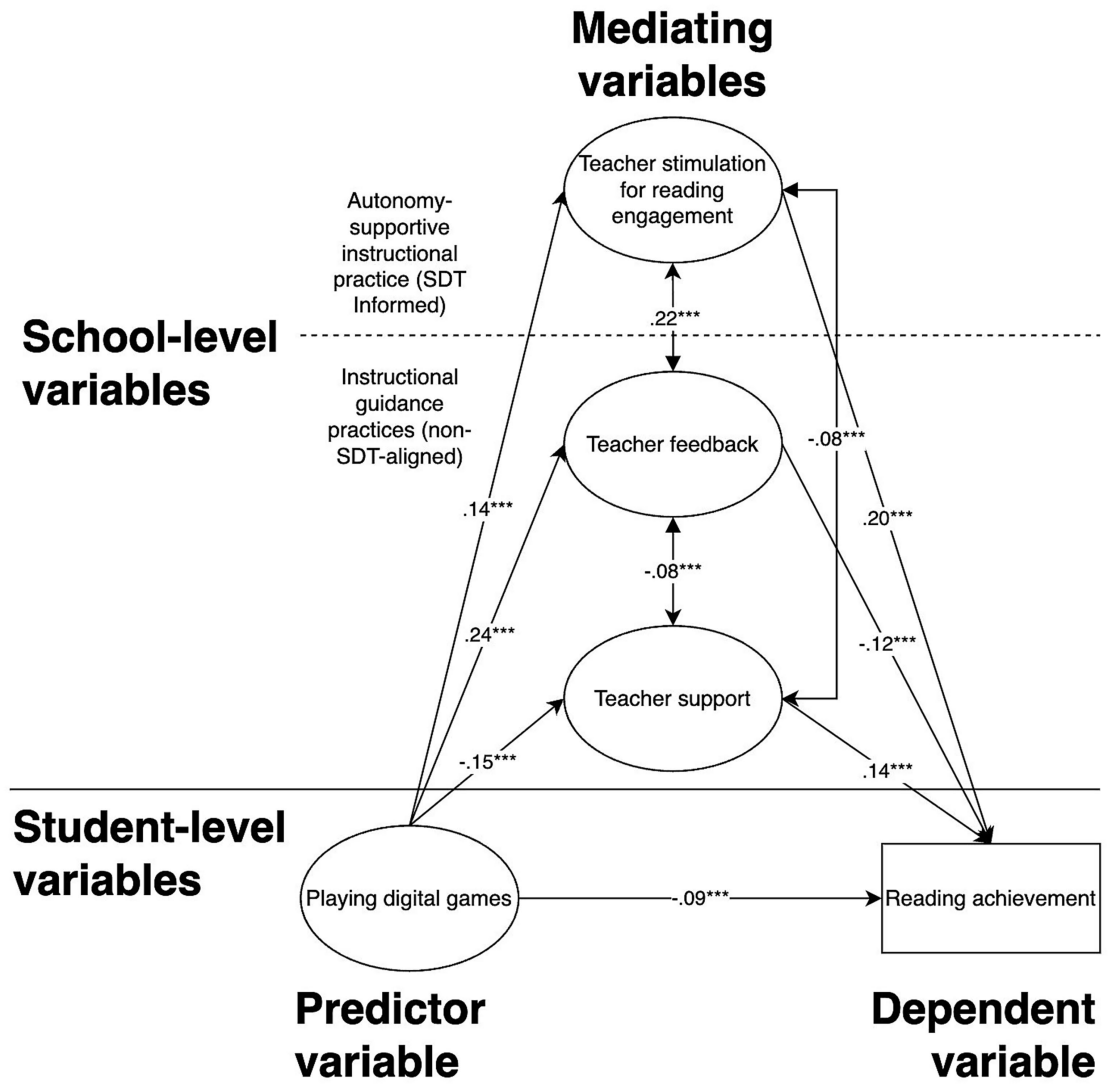
Results for MLSEM (*p < 0.05; **p < 0.01; ***p < 0.001).

The MLSEM model demonstrated good fit: CFI = 0.97, TLI = 0.96, and RMSEA = 0.046. All reported coefficients are based on plausible-value-specific two-level models pooled according to Rubin’s rules, with school-based clustering and normalized student sampling weights. These values exceed conventional thresholds for good fit, indicating that the model adequately represents the relationships among extracurricular digital gaming, school-level instructional practices, and reading achievement across countries (Hu and Bentler, 1999; Xia and Yang, 2019). Standardized path coefficients are presented in Figure 2.

As shown in Figure 2, the results indicate that the negative effect of digital game-playing behavior on reading achievement decreases in magnitude (*β* = −0.09, *p* < 0.001, 95% CI [−0.093, −0.087]). All three school-level instructional mediators were significantly associated with reading achievement: teacher feedback (*β* = −0.12, *p* < 0.001, 95% CI [−0.158, −0.089]), teacher stimulation for reading engagement (*β* = 0.20, *p* < 0.001, 95% CI [0.168, 0.234]), and teacher support (*β* = 0.15, *p* < 0.001, 95% CI [0.107, 0.168]). These coefficients reflect the distinct instructional roles of the three practices, with stimulation showing the strongest positive association with reading performance.

Digital gaming behavior was positively associated with teacher feedback (*β* = 0.24, *p* < 0.001, 95% CI [0.222, 0.262]) and stimulation (*β* = 0.14, *p* < 0.001, 95% CI [0.119, 0.159]), but negatively associated with teacher support (*β* = −0.15, *p* < 0.001, 95% CI [−0.171, −0.133]). At the between-school level, these coefficients indicate contextual covariation within schools’ instructional environments rather than direct effects of an individual student’s gaming behavior on instructional practices. Accordingly, schools differing in digital game-playing behavior also differed modestly in their reported instructional context.

Table 2 indicates that school-level instructional practices contribute to explaining variation in reading achievement beyond student-level differences. Teacher stimulation demonstrates the strongest positive association with reading performance, whereas teacher feedback is negatively associated with reading when modeled at the school level. Similar fit indices across levels indicate that the inclusion of school-level variables improves explanatory power without altering the overall structural integrity of the model. Table 3 summarizes the indirect effects of student-perceived instructional practices, indicating their role as between-school contextual pathways linking PDG and reading achievement.

**Table 2:** Fit indices and standardized regression coefficients for student- and school-level analysis.

Model level	CFI	TLI	RMSEA	PDG → RA (95% CI)	PDG → TF (95% CI)	PDG → TSRE (95% CI)	PDG → TS (95% CI)	TF → RA (95% CI)	TSRE → RA (95% CI)	TS → RA (95% CI)
Level 1 (student-level)	0.97	0.96	0.048	*β* = −0.21, *p* < 0.001, [−0.232, −0.196]	–	–	–	–	–	–
Level 2 (school-level MLSEM)	0.97	0.96	0.046	*β* = −0.09, *p* < 0.001, [−0.093, −0.087]	*β* = 0.24, *p* < 0.001, [0.222, 0.262]	*β* = 0.14, *p* < 0.001, [0.119, 0.159]	*β* = −0.15, *p* < 0.001, [−0.171, −0.133]	*β* = −0.12, *p* < 0.001, [−0.158, −0.089]	*β* = 0.20, *p* < 0.001, [0.168, 0.234]	*β* = 0.15, *p* < 0.001, [0.107, 0.168]

**Table 3:** Results of the mediating effect in the statistical model.

Variables	*β*	95% CIs
PDG-TF-RA	−0.030	[−0.039, −0.021]
PDG-TSRE-RA	0.028	[0.022, 0.034]
PDG-TS-RA	−0.021	[−0.026, −0.016]

PDG, playing digital games; TF, teacher feedback; TSRE, teacher stimulation for reading engagement; TS, teacher support; RA, reading achievement.

Table 3 shows that teacher feedback significantly mediates the association between digital gaming (PDG) and reading achievement (RA), with a negative indirect effect (*β* = −0.030, 95% CI [−0.039, −0.021]). This result indicates that, within the estimated school-level pathway, higher levels of perceived feedback in schools with greater digital gaming were associated with lower reading achievement. Given that PISA’s feedback construct reflects corrective, performance-oriented guidance, this negative indirect path suggests that structured feedback may function differently from autonomy-supportive instructional practices in the instructional context examined here.

Secondly, the mediation effect of teacher support on reading achievement was negative but minimal (*β* = −0.021, 95% CI [−0.026, −0.016]), suggesting that it plays a limited role in explaining the relationship between digital gaming and reading achievement. Although teacher support is commonly associated with positive learning environments, the present findings indicate that school-level variation in perceived support accounted for only a very small portion of the association between digital gaming and reading achievement.

Lastly, teacher stimulation for reading engagement significantly mediates the relationship between digital gaming and reading achievement with a positive indirect effect (*β* = 0.028, 95% CI [0.022, 0.034]). This suggests that autonomy-supportive instructional practices—such as encouraging interpretation, discussion, and personal relevance—may help buffer the negative association between gaming and reading performance, consistent with SDT’s emphasis on the motivational benefits of autonomy-supportive teaching. When the mediating variables are considered collectively, teacher stimulation for reading engagement shows a positive indirect effect, whereas teacher feedback and teacher support show negative indirect effects. Taken together, these findings are broadly consistent with the study’s theoretical distinction between autonomy-supportive and more structurally oriented instructional practices.

A gender-adjusted robustness check yielded a highly similar pattern of results (Supplementary Table 8). The direct association between digital gaming and reading achievement remained virtually unchanged at both the within-school and between-school levels, and the indirect effects through teacher feedback, teacher stimulation for reading engagement, and teacher support retained the same direction, with only modest attenuation in magnitude after gender adjustment. These findings indicate that the substantive conclusions were robust to gender adjustment.

## 5 Discussion

This study examined the structural relationships among extracurricular digital gaming behavior, student-perceived instructional practices, and reading achievement using PISA 2018 data from 79 countries. The results confirmed that extracurricular digital gaming was negatively associated with reading performance while also showing systematic associations with student-perceived instructional practices—positively with teacher feedback and teacher stimulation for reading engagement, and negatively with teacher support. The three instructional practices showed distinct associations with reading achievement: teacher stimulation for reading engagement and teacher support were positively associated with reading achievement, whereas teacher feedback was negatively associated with reading achievement. However, only teacher stimulation for reading engagement functioned as a positive mediator, suggesting that autonomy-supportive practices may help offset the negative association between digital gaming and reading achievement. In contrast, the negative mediation effects of teacher feedback and teacher support suggest that these more structured practices operate differently from autonomy-supportive stimulation within the estimated contextual pathways linking extracurricular digital gaming and reading achievement. Taken together, these indirect patterns are best interpreted as contextual school-level associations rather than as evidence of individual-level motivational mediation. Whereas previous research has focused primarily on gaming’s direct academic associations (Barz et al., 2024; Kuo et al., 2025), the present study shows that school-level instructional practices help explain part of the association between students’ out-of-school digital behaviors and reading achievement across schools.

Framing these results through self-determination theory (SDT) provides a deeper understanding of how digital gaming and reading achievement are connected to instructional contexts. Teacher stimulation for reading engagement reflects a more autonomy-supportive instructional context by encouraging active, self-regulated participation, and its effect may be strengthened when it resonates with motivational patterns linked to digital gaming (Zhang H. et al., 2024; Zhang S. et al., 2024). Many games involve narrative interpretation, problem-solving, and decision-making, which may facilitate reading persistence when instructional environments allow students similar autonomy and engagement (Lai, 2023; Ostovar-Namaghi et al., 2024). Such gameplay mechanics mirror academic reading demands and offer opportunities to align gaming behaviors with classroom reading strategies. Within this SDT-informed framework, the findings are interpreted in relation to differences in instructional context rather than as direct measures of students’ motivational processes (Chiu et al., 2024).

The negative mediation effects of teacher feedback and teacher support warrant careful interpretation, especially because teacher support showed a positive direct association with reading achievement whereas teacher feedback showed a negative direct association. Although both practices traditionally promote competence and relatedness (Yang et al., 2021), their structured, curriculum-oriented nature may contrast with the autonomy-driven responsiveness characteristic of digital gaming (Kokandy, 2021). One possible interpretation is that structured classroom feedback may be less closely aligned with the engagement features commonly associated with digital gaming, such as immediacy, iteration, and rapid response cycles (Carless and Winstone, 2023). This mismatch may help explain the negative mediation patterns and suggests that instructional practices function differently within the school-level association between extracurricular digital gaming and reading achievement.

At the same time, these negative indirect effects should not be attributed to misalignment alone. Given the cross-sectional design and the contextual nature of the school-level measures, alternative explanations remain plausible. For example, reverse causality may be operating, such that lower-performing students or schools receive more frequent feedback or support (Chen and Lin, 2025). In addition, unmeasured confounders—such as socioeconomic composition, prior achievement, or other features of school quality—may have influenced both instructional patterns and reading outcomes (Andrade, 2024). Cultural heterogeneity may also matter: although the measures demonstrated configural, metric, and scalar invariance across the classification systems, feedback and support may still differ in their practical meaning, tone, and perceived value across educational contexts (Deffner et al., 2022). Accordingly, the negative indirect effects of teacher feedback and teacher support are best interpreted as suggestive contextual patterns rather than definitive evidence of a single underlying mechanism.

To bridge the gap between students’ gaming-influenced engagement patterns and classroom instruction, feedback and support strategies may benefit from greater autonomy sensitivity and responsiveness (Dahalan et al., 2024; Howard et al., 2024). Digital games provide immediate, specific, and actionable feedback that supports self-regulation (Liu and Hwang, 2024), whereas classroom feedback often emphasizes correction and evaluation (Fu and Li, 2024). Instructional approaches that emulate the interactive qualities of digital environments—and position teacher support as collaborative rather than directive—may offer one way of responding to these patterns, particularly for digitally engaged learners (Yang et al., 2024). To clarify the interpretation of the findings, the discussion below is organized under two thematic headings: (1) associations with reading achievement and (2) the mediating role of instructional practices in the relationship between extracurricular digital gaming and reading achievement.

### 5.1 Direct associations with reading achievement

Digital game-playing behavior was negatively associated with reading achievement (H1), while two instructional practices—teacher stimulation for reading engagement and teacher support—showed positive direct associations with reading performance (H2b, H4b). Teacher feedback, however, demonstrated a negative direct association with reading achievement (H3b), indicating that higher aggregated levels of corrective, performance-oriented feedback at the school level correspond to lower reading performance. Among these practices, teacher stimulation for reading engagement appears most favorable, as it is more consistent with self-directed literacy engagement and intrinsic motivation (Ryan and Deci, 2020).

The negative association between digital gaming behavior and reading achievement may stem from several factors, including compulsive gaming tendencies, gameplay content, and design features (Borgonovi, 2016; Behl et al., 2024). Rather than being motivated solely by enjoyment, problematic or excessive gaming is characterized by persistent engagement despite negative academic, social, or psychological consequences (Weinstein, 2010; Yilmaz and Tunca, 2024). Because digital gaming typically occurs outside the classroom, its timing may interfere with study routines or activities that support reading development (Zhang and Gu, 2022). Moreover, depending on genre and platform, many games lack text-rich environments conducive to literacy growth (Apperley and Walsh, 2012). The persistence of this negative association—even after accounting for school-level instructional practices—underscores the need to examine how extracurricular gaming interacts with literacy development (Fu et al., 2024). That this negative relationship appears across the pooled international sample highlights a global challenge for educators and policymakers.

Within SDT, stimulation for reading engagement can be understood as a more autonomy-supportive instructional practice that encourages interpretive thinking and self-directed participation (Zhang H. et al., 2024; Zhang S. et al., 2024), thereby helping to explain why its association with reading achievement differs from those of feedback and support. In contrast, feedback and support showed less favorable indirect patterns in the present model, which may help explain why the indirect associations varied across the instructional practices examined. When classroom feedback is experienced as prescriptive or corrective rather than informational, it may undermine students’ sense of competence (Ryan and Deci, 2017, 2020). Similarly, support that emphasizes guidance without offering autonomy may be perceived as less relevant by students accustomed to choice-driven, self-paced digital environments. These patterns are best understood in relation to differences in instructional context, with SDT offering a theoretical lens for interpreting their motivational relevance.

Gamification offers an alternative to traditional instruction by incorporating game elements into reading activities to enhance motivation and engagement (Dahalan et al., 2024; Fu et al., 2024). Embedding mechanics such as progression levels, experience points, and digital badges can encourage students to persist with challenging texts by linking achievement to comprehension milestones (Cigdem et al., 2024; Li X. et al., 2024; Liu et al., 2024). Interactive storytelling formats—where student choices shape the narrative—promote critical thinking and deepen reading comprehension (Li and Chu, 2021; Tanouri et al., 2024). Collaborative reading quests further enhance social engagement and intrinsic motivation by fostering teamwork around literacy goals (Manzano León et al., 2022). The effectiveness of such approaches, however, depends on teachers’ ability to align game-informed practices with meaningful literacy goals rather than applying game elements superficially.

### 5.2 The indirect effect of playing digital games on reading achievement via student-perceived instructional practices

These findings highlight teacher stimulation for reading engagement as the instructional practice associated with the most favorable indirect pathway between extracurricular digital gaming and reading achievement, thereby partially counterbalancing the negative association between digital gaming and reading achievement. Viewed together, the indirect paths suggest that this autonomy-supportive practice was more favorably aligned with reading achievement than the more structured forms of feedback and support in the present model (Reeve, 2012; Ryan and Deci, 2020). This underscores the need to align instructional practices more closely with autonomy-supportive structures familiar in gaming environments (Jaramillo-Mediavilla et al., 2024). Among the three mediators, teacher stimulation appears most consistent with promoting intrinsic motivation and positioning literacy as an engaging, self-directed activity (Weinstein, 2010; Ryan and Deci, 2017). The effectiveness of instructional practices may therefore depend on the extent to which they reflect more autonomy-supportive or more structurally oriented forms of instructional context in relation to extracurricular digital gaming (Guthrie et al., 2006; Ho and Lau, 2018).

Teacher feedback and support show a nuanced pattern: although teacher support displays a positive direct association with reading achievement and teacher feedback a negative direct association, their indirect effects suggest that these practices function differently from teacher stimulation for reading engagement within the estimated school-level pathways (Wu et al., 2021; Cattoni et al., 2024). In practice, adopting game-inspired strategies—such as real-time progress tracking, challenge-based assessments, and collaborative support—can transform these practices into autonomy- and competence-supportive tools (Boubakri and Nafil, 2024; Cattoni et al., 2024). Reframing teacher support as collaborative rather than directive may also increase its motivational relevance, particularly for students accustomed to high levels of self-direction in gaming environments (Qiao et al., 2022). These insights point to the need for more nuanced applications of feedback and support in classrooms where gaming behaviors are widespread.

As classroom observers and facilitators, teachers can mitigate the negative influence of excessive gaming by adapting instructional strategies to better reflect students’ digital engagement patterns (Sivakumar, 2024). More flexible and responsive feedback systems may convert challenges into competence-enhancing opportunities (Yang et al., 2021; Ma et al., 2023). In contrast, rigid, paper-based reading instruction that does not acknowledge digital forms of literacy may risk reinforcing disengagement (Behl et al., 2024; Díaz et al., 2024). Integrating teacher-guided digital tools—such as interactive reading platforms, narrative-based tasks, and choice-driven comprehension activities—offers an approach aligned with SDT’s emphasis on autonomy and competence (Sofiana and Mubarok, 2020; Zhang and Gu, 2022). By bridging traditional literacy instruction with students’ digital learning preferences, teachers may foster intrinsic motivation for reading without relying on inflexible instructional routines.

When thoughtfully applied, gamification can transform literacy tasks into engaging, structured experiences aligned with students’ gaming motivations. To be effective, however, such approaches must promote intrinsic motivation rather than rely solely on external rewards (Balalle, 2024; Hong et al., 2024). Achievement mechanics—such as badges, narrative quests, and collaborative challenges—can support literacy when they address students’ needs for autonomy, competence, and relatedness (Cattoni et al., 2024; Li L. et al., 2024; Li X. et al., 2024). This approach aligns with SDT by promoting agency, meaningful engagement, and sustained challenge within teacher-facilitated instruction (Chavez and Palaoag, 2024; Chiu et al., 2024). Nonetheless, superficial or reward-heavy gamification may undermine deep engagement (Matallaoui et al., 2017; Dah et al., 2024). When strategically implemented, teacher-led gamification may help reposition gaming as a motivational resource rather than a potential barrier to literacy development (Marzal and Cardama, 2021; Lyu, 2024; Ng et al., 2024).

## 6 Conclusion

This study offers new insights into the complex relationship between digital gaming, student-perceived instructional practices, and reading achievement, grounded in self-determination theory (SDT). Using data from PISA 2018, the analysis demonstrates that while digital gaming is negatively associated with reading performance, this association is not uniform across instructional contexts. School-level instructional practices—teacher feedback, teacher stimulation for reading engagement, and teacher support—relate to reading achievement in distinct ways and help clarify how classroom climates intersect with students’ extracurricular gaming behaviors. Among these practices, teacher stimulation for reading engagement stands out as the only instructional practice associated with a positive indirect pathway linking digital gaming and reading achievement, thereby mitigating part of the negative gaming–reading association. More precisely, the results indicate that student-perceived instructional practices function as contextual school-level pathways that help explain part of the association between extracurricular digital gaming and reading achievement.

The findings further indicate that the negative indirect patterns involving feedback and support require cautious interpretation, as they may reflect multiple contextual processes rather than a single explanatory mechanism. These results underscore the importance of distinguishing between autonomy-supportive and non–autonomy-supportive instructional practices rather than treating teacher practices as motivationally uniform. Therefore, the findings suggest that the association between extracurricular digital gaming and reading achievement is not uniform across schools but varies with the motivational character of the instructional environment.

Taken together, the findings suggest that literacy instruction may benefit from approaches that integrate autonomy-supportive, game-informed features capable of resonating with digitally engaged learners. Rather than framing digital gaming solely as a threat to reading development, educators may reconceptualize it as a contextual factor whose negative associations can be mitigated through instructional practices that promote choice, relevance, and self-directed engagement. Designing literacy environments that reflect the motivational dynamics familiar to digitally immersed students may foster more sustainable reading habits and support academic engagement in contemporary learning contexts.

## 7 Limitations, implications, and future research

Despite offering valuable insights, this study has limitations that affect generalizability. First, the reliance on self-reported data introduces the potential for response bias (Rosenman et al., 2011). Students’ perceptions may not fully reflect actual classroom practices or their underlying motivational experiences. This constrains the precision with which autonomy, competence, and relatedness can be inferred from instructional environments. To enhance validity, future research should combine self-report data with objective measures, such as teacher observations or digital activity logs, to better capture students’ motivational patterns across learning contexts.

Second, PISA 2018 omits forms like mobile or educational games, which may influence literacy development (Gros, 2007; Sofiana and Mubarok, 2020). This limits assessment of how educational games aligned with instruction affect engagement and achievement. Research suggests that open-ended, collaborative, or narrative-based games may foster intrinsic motivation for literacy tasks (Hwang et al., 2024), whereas highly structured or competitive games may promote extrinsic goals that are less effective for sustained reading engagement (Alzahrani and Griffiths, 2024). Due to data constraints, the potential instructional value of educational gaming could not be examined. Future PISA cycles should broaden their scope to capture a wider range of gaming behaviors, enabling a more comprehensive understanding of their academic impact.

Third, PISA’s cross-sectional design limits causal inference (Van Der Stede, 2014). We cannot determine whether autonomy-supportive teacher behaviors lead to greater reading engagement, or if students predisposed to autonomous learning are more responsive to such practices. These correlational findings identify key areas for further study. Longitudinal studies are needed to examine how gaming behaviors, instructional practices, and reading achievement evolve over time and to clarify potential causal pathways. Relatedly, because the mediating variables were modeled as school-level indicators derived from student perceptions, the study does not test individual-level motivational mediation (OECD, 2018, 2019c). Accordingly, the findings should not be interpreted as showing that individual students’ autonomy, competence, or relatedness directly mediated the relationship between digital gaming and reading achievement. This limitation is especially important for interpreting the negative indirect effects of teacher feedback and teacher support, because reverse causality cannot be ruled out; lower-performing students or schools may attract more corrective feedback or support rather than feedback or support producing lower achievement (Chen and Lin, 2025). Likewise, the model did not include potentially important confounders—such as socioeconomic composition, prior achievement, or broader indicators of school quality—that may shape both instructional practices and reading outcomes (Andrade, 2024). Future research should examine student-level mediation and cross-level moderation using direct motivational measures and classroom-level or longitudinal designs.

Fourth, the PISA sampling design introduces interpretive limitations for certain countries, such as Canada, where results must be viewed within the context of national sampling constraints (Phillips, 2024). This highlights the need for culturally sensitive interpretation. Educational systems vary in how they emphasize autonomy, competence, and relatedness, which may shape student responses to teacher feedback and support in literacy and gaming contexts (Howard et al., 2024). Although variation was addressed using CNT, WCR, and TTS, future work should examine how policy and culture shape motivational processes in gaming-influenced reading development. More specifically, even when constructs are statistically comparable across groups, the lived meaning and classroom function of feedback and support may still differ across national and cultural contexts.

Fifth, gender remains an important consideration in interpreting the negative link between gaming and reading. Prior PISA findings show that boys typically game more and perform lower in reading than girls (OECD, 2019a). In response to this concern, we conducted a gender-adjusted robustness check, and the overall pattern of direct and indirect effects remained substantively stable (Supplementary Table 8). Nevertheless, gender may still shape how digital gaming relates to reading achievement and instructional pathways in more nuanced ways than can be captured by a single adjustment term. Future studies should therefore examine gender not only as a control variable but also as a potential moderator of the gaming–reading relationship and of the instructional mediation pathways.

Sixth, although the instructional-practice measures demonstrated configural, metric, and scalar invariance across CNT, WCR, and TTS in the present study, cross-national differences in the lived meaning and classroom enactment of these practices may remain. Statistical invariance supports comparability of measurement, but it does not eliminate the possibility that teacher feedback, teacher support, and teacher stimulation for reading engagement carry somewhat different pedagogical connotations across educational systems and cultural contexts (Muthén and Asparouhov, 2018; Robitzsch and Lüdtke, 2023). Accordingly, future research should complement invariance testing with country-specific or culturally responsive analyses to better capture cross-national heterogeneity in how students interpret these instructional practices. Beyond these limitations, the study also offers theoretical, practical, and policy-relevant implications for literacy instruction in digitally saturated learning environments.

This study extends self-determination theory by showing how student-perceived instructional practices function as contextual school-level pathways linking digital gaming and literacy development. It provides empirical evidence that autonomy-supportive reading stimulation may buffer gaming’s negative associations, while more traditional forms of feedback and support may be associated with less favorable indirect patterns in the present model. Teacher strategies that foster autonomy are therefore essential in addressing gaming-related challenges. These findings not only contribute to SDT-informed educational research but also offer practical guidance for educators seeking to leverage digital tools to support reading achievement.

Beyond theoretical contributions, the study offers practical guidance for educators and policymakers seeking to improve reading instruction in digitally saturated environments. Professional development should prioritize autonomy-supportive teaching strategies that integrate game-based elements aligned with students’ intrinsic needs for autonomy, competence, and relatedness (Li L. et al., 2024). When literacy goals are framed within engaging, game-like structures, student motivation and learning outcomes improve (Villalta et al., 2011). Educational policy should support teachers in adapting feedback and support practices to prevent competence frustration and reinforce self-directed learning. Immediate, formative tools—such as scaffolded digital platforms (e.g., ReadTheory, Epic!, and CommonLit)—can help students self-regulate through game-like feedback mechanisms (Wood, 2021; Lo-Philip, 2024).

Future instructional strategies should prioritize teacher-guided implementations of game-based features—such as progressive reading tasks, real-time feedback, and interactive storytelling—that reinforce autonomy and competence. Instead of generic rewards, educators can embed narrative-driven challenges that reflect students’ digital engagement patterns (Jaramillo-Mediavilla et al., 2024; Li L. et al., 2024). This approach transforms digital distractions into meaningful literacy interventions that enhance engagement while maintaining instructional coherence. To support these efforts, policymakers should promote curricula that incorporate game-based learning and invest in large-scale data collection—such as through PISA—to inform evidence-based decisions on digital literacy integration.

Future research should further examine how cultural context shapes the role of instructional practices in the relationship between digital gaming and educational outcomes. As SDT suggests, the relative importance of autonomy, competence, and relatedness varies across cultures (Howard et al., 2024; Lo, 2024). Students in collectivist societies may respond differently to feedback and support than those in individualist contexts. Cultural norms around education and gaming may therefore shape these dynamics, reinforcing the need for contextually responsive teaching.

## Data Availability

The original contributions presented in the study are included in the article/Supplementary material, further inquiries can be directed to the corresponding author.

## References

[ref1] Acun Çelik S. Özkan Elgün İ. Kalelioğlu F. (2024). Assessment of student ICT competence according to mathematics, science, and reading literacy: evidence from PISA 2018. Large Scale Assess. Educ. 12:30. doi: 10.1186/s40536-024-00218-7

[ref2] Ali S. I. Rogers M. L. Schatschneider C. Joiner T. E. Keel P. K. (2026). Establishing the measurement invariance of the eating disorder inventory across Hispanic white, non-Hispanic Asian, non-Hispanic black or African American, and non-Hispanic white adults. Psychol. Assess. 17:453. doi: 10.1037/pas0001453, 41505327 PMC12910446

[ref3] Alzahrani A. K. D. Griffiths M. D. (2024). Problematic gaming and students’ academic performance: a systematic review. Int. J. Ment. Health Addiction 23, 4062–4095. doi: 10.1007/s11469-024-01338-5

[ref4] Amzalag M. Kadusi D. Peretz S. (2024). Enhancing academic achievement and engagement through digital game-based learning: an empirical study on middle school students. J. Educ. Comput. Res. 62, 1209–1233. doi: 10.1177/07356331241236937

[ref5] Andrade C. (2024). Confounding by indication, confounding variables, covariates, and independent variables: knowing what these terms mean and when to use which term. Indian J. Psychol. Med. 46, 78–80. doi: 10.1177/02537176241227586, 38524951 PMC10958068

[ref6] Anjum R. Nodi N. H. Das P. R. Roknuzzaman A. S. M. Sarker R. Islam M. R. (2024). Exploring the association between online gaming addiction and academic performance among the school-going adolescents in Bangladesh: a cross-sectional study. Health Sci. Rep. 7, 1–19. doi: 10.1002/hsr2.70043, 39221049 PMC11362216

[ref7] Anthony W. L. Zhu Y. Nower L. (2021). The relationship of interactive technology use for entertainment and school performance and engagement: evidence from a longitudinal study in a nationally representative sample of middle school students in China. Comput. Human Behav. 122, 1–9. doi: 10.1016/j.chb.2021.106846

[ref8] Aparicio J. Cordero J. M. Ortiz L. (2021). Efficiency analysis with educational data: how to deal with plausible values from international large-scale assessments. Mathematics 9, 1–16. doi: 10.3390/math9131579

[ref9] Apperley T. Walsh C. (2012). What digital games and literacy have in common: a heuristic for understanding pupils’ gaming literacy. Literacy 46, 115–122. doi: 10.1111/j.1741-4369.2012.00668.x

[ref10] Asrifan A. Kaddas B. Mulyadi M. Pratiwi W. R. Supriadi S. Jabu B. (2024). “Cross-cultural gamification: understanding design and user experience in different cultural contexts,” in Advances in Media, Entertainment, and the Arts, eds. Marcão R. Ribeiro Santos V. (Hershey, PA, USA: IGI Global), 1–28.

[ref11] Balalle H. (2024). Exploring student engagement in technology-based education in relation to gamification, online/distance learning, and other factors: a systematic literature review. Soc. Sci. Humanit. Open 9, 1–10. doi: 10.1016/j.ssaho.2024.100870

[ref12] Banerjee R. Halder S. (2021). Amotivation and influence of teacher support dimensions: a self-determination theory approach. Heliyon 7, 1–11. doi: 10.1016/j.heliyon.2021.e07410, 34278021 PMC8264603

[ref13] Barrett P. (2007). Structural equation modelling: adjudging model fit. Pers. Individ. Differ. 42, 815–824. doi: 10.1016/j.paid.2006.09.018

[ref14] Barz N. Benick M. Dörrenbächer-Ulrich L. Perels F. (2024). The effect of digital game-based learning interventions on cognitive, metacognitive, and affective-motivational learning outcomes in school: a meta-analysis. Rev. Educ. Res. 94, 193–227. doi: 10.3102/00346543231167795

[ref15] Basaran B. Simsek Ö. (2024). Examination of gender-based video game-playing classes: influencing determinants and relations to academic achievement. Comput. Assist. Learn. 40, 2574–2588. doi: 10.1111/jcal.12920

[ref16] Baysu G. Agirdag O. De Leersnyder J. (2023). The association between perceived discriminatory climate in school and student performance in math and reading: a cross-national analysis using PISA 2018. J. Youth Adolesc. 52, 619–636. doi: 10.1007/s10964-022-01712-3, 36477568 PMC9884186

[ref17] Beauducel A. Herzberg P. Y. (2006). On the performance of maximum likelihood versus means and variance adjusted weighted least squares estimation in CFA. Struct. Equ. Model. 13, 186–203. doi: 10.1207/s15328007sem1302_2

[ref18] Behl A. Jayawardena N. Bhardwaj S. Pereira V. Del Giudice M. Zhang J. (2024). Examining the failure of gamification in implementing innovation from the perspective of problematization in the retail sectors of emerging economies. Technovation 129, 1–13. doi: 10.1016/j.technovation.2023.102902

[ref19] Bhagat S. Jeong E. J. Kim D. J. (2020). The role of individuals’ need for online social interactions and interpersonal incompetence in digital game addiction. Int. J. Hum. Comput. Interact. 36, 449–463. doi: 10.1080/10447318.2019.1654696

[ref20] Blume C. (2020). Games people (don’t) play: an analysis of pre-service EFL teachers’ behaviors and beliefs regarding digital game-based language learning. Comput. Assist. Lang. Learn. 33, 109–132. doi: 10.1080/09588221.2018.1552599

[ref21] Borgonovi F. (2016). Video gaming and gender differences in digital and printed reading performance among 15-year-olds students in 26 countries. J. Adolesc. 48, 45–61. doi: 10.1016/j.adolescence.2016.01.004, 26874783

[ref22] Boubakri M. Nafil K. (2024). Gamification solutions for persons with disabilities: a systematic literature review. Univ. Access Inf. Soc. 24:1009. doi: 10.1007/s10209-024-01170-7

[ref23] Brown G. T. L. Harris L. R. O’Quin C. Lane K. E. (2017). Using multi-group confirmatory factor analysis to evaluate cross-cultural research: identifying and understanding non-invariance. Int. J. Res. Method Educ. 40, 66–90. doi: 10.1080/1743727X.2015.1070823

[ref24] Buuren S. V. Groothuis-Oudshoorn K. (2011). Mice: multivariate imputation by chained equations in R. J. Stat. Softw. 45, 1–67. doi: 10.18637/jss.v045.i03

[ref25] Cabellos B. Sánchez D. L. Pozo J.-I. (2023). Do future teachers believe that video games help learning? Tech. Know. Learn. 28, 803–821. doi: 10.1007/s10758-021-09586-3

[ref26] Candel E. C. de-la-Peña C. Yuste B. C. (2024). Pre-service teachers’ perception of active learning methodologies in history: flipped classroom and gamification in an e-learning environment. Educ. Inf. Technol. 29, 3365–3387. doi: 10.1007/s10639-023-11924-0

[ref27] Carless D. Winstone N. (2023). Teacher feedback literacy and its interplay with student feedback literacy. Teach. High. Educ. 28, 150–163. doi: 10.1080/13562517.2020.1782372

[ref28] Cattoni A. Anderle F. Venuti P. Pasqualotto A. (2024). How to improve reading and writing skills in primary schools: a comparison between gamification and pen-and-paper training. Int. J. Child Comput. Interact. 39, 1–13. doi: 10.1016/j.ijcci.2024.100633

[ref29] Chavez O. J. Palaoag T. (2024). AI-driven mobile application: unraveling students’ motivational feature preferences for reading comprehension. JRIT 17, 226–242. doi: 10.1108/JRIT-02-2024-0045

[ref30] Chen Y. Lin S.-W. (2025). Paradoxical associations between support structures and achievement: a cross-national exploratory analysis of Taiwan and Finland using PISA 2022. Discov. Educ. 4, 1–37. doi: 10.1007/s44217-025-00957-x

[ref31] Chiu T. K. F. Falloon G. Song Y. Wong V. W. L. Zhao L. Ismailov M. (2024). A self-determination theory approach to teacher digital competence development. Comput. Educ. 214, 1–14. doi: 10.1016/j.compedu.2024.105017

[ref32] Chu S. Hwang G. Chien S. Chang S. (2023). Incorporating teacher intelligence into digital games: an expert system-guided self-regulated learning approach to promoting students’ performance in digital gaming contexts. Br. J. Educ. Technol. 54, 534–553. doi: 10.1111/bjet.13260

[ref33] Cigdem H. Ozturk M. Karabacak Y. Atik N. Gürkan S. Aldemir M. H. (2024). Unlocking student engagement and achievement: the impact of leaderboard gamification in online formative assessment for engineering education. Educ. Inf. Technol. 29, 24835–24860. doi: 10.1007/s10639-024-12845-2

[ref34] Clark D. A. Bowles R. P. (2018). Model fit and item factor analysis: Overfactoring, underfactoring, and a program to guide interpretation. Multivar. Behav. Res. 53, 544–558. doi: 10.1080/00273171.2018.1461058, 29683723

[ref35] Cole C. Parada R. H. Mackenzie E. (2024). A scoping review of video games and learning in secondary classrooms. J. Res. Technol. Educ. 56, 544–577. doi: 10.1080/15391523.2023.2186546

[ref37] Crawford G. Brock T. (2024). Just fun and games? A sociological consideration of fun in video games. Games Cult. 2024, 1–17. doi: 10.1177/15554120241254876

[ref38] Dah J. Hussin N. Zaini M. K. Isaac Helda L. Senanu Ametefe D. Adozuka Aliu A. (2024). Gamification is not working: why? Games Cult. 20, 934–957. doi: 10.1177/15554120241228125

[ref39] Dahalan F. Alias N. Shaharom M. S. N. (2024). Gamification and game based learning for vocational education and training: a systematic literature review. Educ. Inf. Technol. 29, 1279–1317. doi: 10.1007/s10639-022-11548-w, 36688221 PMC9838474

[ref40] Davis T. Jackson E. (2013). Bookworm: Ultimate guide to the Award Winning game. North Charleston: CreateSpace Independent Publishing Platform.

[ref41] De Naeghel J. Van Keer H. Vansteenkiste M. Rosseel Y. (2012). The relation between elementary students’ recreational and academic reading motivation, reading frequency, engagement, and comprehension: a self-determination theory perspective. J. Educ. Psychol. 104, 1006–1021. doi: 10.1037/a0027800

[ref42] Deci E. L. Ryan R. M. (2012) “Self-determination theory,” Handbook of Theories of social Psychology: Volume I Lange P. A. M. Van Kruglanski A. W. Higgins E. T. Thousand Oaks SAGE Publications 416–437

[ref43] Deffner D. Rohrer J. M. McElreath R. (2022). A causal framework for cross-cultural generalizability. Adv. Methods Pract. Psychol. Sci. 5, 1–18. doi: 10.1177/25152459221106366

[ref44] Díaz B. Nussbaum M. Greiff S. Santana M. (2024). The role of technology in reading literacy: is Sweden going back or moving forward by returning to paper-based reading? Comput. Educ. 213, 1–12. doi: 10.1016/j.compedu.2024.105014

[ref45] Dietrich J. Schmiedek F. Moeller J. (2022). Academic motivation and emotions are experienced in learning situations, so let’s study them. Introduction to the special issue. Learn. Instr. 81, 1–4. doi: 10.1016/j.learninstruc.2022.101623

[ref46] ed. Dillon R. (2020). The Digital Gaming Handbook. Boca Raton: CRC Press.

[ref47] Dong N. Reinke W. M. Herman K. C. Bradshaw C. P. Murray D. W. (2016). Meaningful effect sizes, intraclass correlations, and proportions of variance explained by covariates for planning two- and three-level cluster randomized trials of social and behavioral outcomes. Eval. Rev. 40, 334–377. doi: 10.1177/0193841X16671283, 27694127

[ref48] Drummond A. Sauer J. D. (2020). Timesplitters: playing video games before (but not after) school on weekdays is associated with poorer adolescent academic performance. A test of competing theoretical accounts. Comput. Educ. 144, 1–12. doi: 10.1016/j.compedu.2019.103704

[ref50] Ebadi S. Ahmadi R. (2024). A native video gamer’s journey toward multi-literacy development: a narrative inquiry. J. Lang. Identity Educ. 23, 675–688. doi: 10.1080/15348458.2022.2029450

[ref52] Elson M. Ferguson C. J. (2014). Twenty-five years of research on violence in digital games and aggression: empirical evidence, perspectives, and a debate gone astray. Eur. Psychol. 19, 33–46. doi: 10.1027/1016-9040/a000147

[ref53] Enders C. K. (2022). Applied Missing data Analysis. 2nd Edn New York: The Guilford Press.

[ref54] Facchino A. P. Marchetti D. Colasanti M. Fontanesi L. Verrocchio M. C. (2025). The use of serious games for psychological education and training: a systematic review. Front. Educ. 10, 1–22. doi: 10.3389/feduc.2025.1511729

[ref55] Faust K. A. Faust D. Meyer J. F. Cook N. E. (2020). “Selecting and refining measures,” in The Oxford Handbook of Digital Technologies and mental Health, eds. Potenza M. N. Faust K. Faust D. (Oxford: Oxford University Press), 144–153.

[ref56] Felicia P. (2009). Digital games in Schools: Handbook for Teachers. Brussels, Belgium: European Schoolnet.

[ref57] Fu M. Li S. (2024). The associations between foreign language anxiety and the effectiveness of immediate and delayed corrective feedback. Foreign Lang. Ann. 57, 201–228. doi: 10.1111/flan.12708

[ref58] Fu K. Liu Z. Ren X. Zhang S. (2024). Design and research of educational mode in context of teaching gamification. Entertain. Comput. 50, 1–10. doi: 10.1016/j.entcom.2024.100685

[ref59] Gamlem S. M. (2015). Feedback to support learning: changes in teachers’ practice and beliefs. Teach. Dev. 19, 461–482. doi: 10.1080/13664530.2015.1060254

[ref61] Garson G. D. (2015). Missing Values Analysis and data Imputation. Asheboro: Statistical Associates Publishing.

[ref62] González-González C. S. Toledo-Delgado P. A. Muñoz-Cruz V. Arnedo-Moreno J. (2022). Gender and age differences in preferences on game elements and platforms. Sensors 22, 1–15. doi: 10.3390/s22093567, 35591266 PMC9101555

[ref63] Grønli K. M. Walgermo B. R. McTigue E. M. Uppstad P. H. (2025). Feedback practices on young students’ oral reading: a systematic review. Rev. Educ. Res. 96, 391–434. doi: 10.3102/00346543241306070

[ref64] Gros B. (2007). Digital games in education: the design of games-based learning environments. J. Res. Technol. Educ. 40, 23–38. doi: 10.1080/15391523.2007.10782494

[ref65] Guthrie J. T. Wigfield A. Humenick N. M. Perencevich K. C. Taboada A. Barbosa P. (2006). Influences of stimulating tasks on reading motivation and comprehension. J. Educ. Res. 99, 232–246. doi: 10.3200/JOER.99.4.232-246

[ref66] Hao Q. SmithIv D. H. Ding L. Ko A. Ottaway C. Wilson J. . (2022). Towards understanding the effective design of automated formative feedback for programming assignments. Comput. Sci. Educ. 32, 105–127. doi: 10.1080/08993408.2020.1860408

[ref68] Henrickson K. Zou Y. Wang Y. (2015). Flexible and robust method for missing loop detector data imputation. Transp. Res. Rec. 2527, 29–36. doi: 10.3141/2527-04

[ref69] Hirschfeld G. von Brachel R. (2014). Improving multiple-group confirmatory factor analysis in R – a tutorial in measurement invariance with continuous and ordinal indicators. Pract. Assess. Res. Eval. 19, 1–12. doi: 10.7275/QAZY-2946

[ref70] Ho E. S. C. Lau K. L. (2017). “Learning strategies, reading engagement, learning environment and students’ reading performance in east Asian societies,” in What We Learned from PISA: The Outstanding Performance of Students in Hong Kong and East Asia, ed. Ho E. S. C. (World Scientific), 263–300.

[ref71] Ho E. S. C. Lau K. (2018). Reading engagement and reading literacy performance: effective policy and practices at home and in school. J. Res. Read. 41, 657–679. doi: 10.1111/1467-9817.12246

[ref72] Hong J. Cheng C. Hwang M. Lee C. Chang H. (2009). Assessing the educational values of digital games. Comput. Assist. Learn. 25, 423–437. doi: 10.1111/j.1365-2729.2009.00319.x

[ref73] Hong Y. Saab N. Admiraal W. (2024). Approaches and game elements used to tailor digital gamification for learning: a systematic literature review. Comput. Educ. 212, 1–21. doi: 10.1016/j.compedu.2024.105000

[ref74] Howard J. L. Slemp G. R. Wang X. (2024). Need support and need thwarting: a meta-analysis of autonomy, competence, and relatedness supportive and thwarting behaviors in student populations. Personal. Soc. Psychol. Bull. 51, 1552–1573. doi: 10.1177/01461672231225364, 38291862 PMC12276404

[ref75] Hu L. Bentler P. M. (1999). Cutoff criteria for fit indexes in covariance structure analysis: conventional criteria versus new alternatives. Struct. Equ. Model. 6, 1–55. doi: 10.1080/10705519909540118

[ref76] Hu J. Yu R. (2021). The effects of ICT-based social media on adolescents’ digital reading performance: a longitudinal study of PISA 2009, PISA 2012, PISA 2015 and PISA 2018. Comput. Educ. 175:104342. doi: 10.1016/j.compedu.2021.104342

[ref77] Huang J. Siu C. T.-S. Cheung H. (2022). Longitudinal relations among teacher-student closeness, cognitive flexibility, intrinsic reading motivation, and reading achievement. Early Child Res. Q. 61, 179–189. doi: 10.1016/j.ecresq.2022.07.009

[ref78] Hwang G.-J. Huang H. Chen H.-Y. (2024). Promoting students’ civic literacy and positive learning behaviors: a supportive feedback-based decision-making gaming approach. Educ. Inf. Technol. 30:227. doi: 10.1007/s10639-024-12839-0

[ref79] IEA (2019). “PIRLS 2021 assessment frameworks,” in PIRLS 2021 Assessment Frameworks. eds. Mullis I. Martin M. O. (Chestnut Hill, MA, USA: International Association for the Evaluation of Educational Achievement).

[ref80] Islam M. I. Biswas R. K. Khanam R. (2020). Effect of internet use and electronic game-play on academic performance of Australian children. Sci. Rep. 10, 21727–21710. doi: 10.1038/s41598-020-78916-9, 33303948 PMC7729852

[ref81] Iurea C. (2015). Classroom environment between stimulation and discouragement. Teacher’s contribution to creating a new socio-affective environment favoring the teacher-student communication. Procedia. Soc. Behav. Sci. 203, 367–373. doi: 10.1016/j.sbspro.2015.08.310

[ref82] Jamshidian M. Jalal S. Jansen C. (2014). MissMech: an R package for testing homoscedasticity, multivariate normality, and missing completely at random (MCAR). J. Stat. Soft. 56, 1–31. doi: 10.18637/jss.v056.i06

[ref83] Jaramillo-Mediavilla L. Basantes-Andrade A. Cabezas-González M. Casillas-Martín S. (2024). Impact of gamification on motivation and academic performance: a systematic review. Educ. Sci. 14, 1–16. doi: 10.3390/educsci14060639

[ref84] Jensen M. T. Solheim O. J. Idsøe E. M. C. (2019). Do you read me? Associations between perceived teacher emotional support, reader self-concept, and reading achievement. Soc. Psychol. Educ. 22, 247–266. doi: 10.1007/s11218-018-9475-5

[ref85] Jeong E. J. Kim D. J. Lee D. M. (2017). Why do some people become addicted to digital games more easily? A study of digital game addiction from a psychosocial health perspective. Int. J. Hum. Comput. Interact. 33, 199–214. doi: 10.1080/10447318.2016.1232908

[ref86] Jeong S. Lee Y. (2019). Consequences of not conducting measurement invariance tests in cross-cultural studies: a review of current research practices and recommendations. Adv. Dev. Hum. Resour. 21, 466–483. doi: 10.1177/1523422319870726

[ref87] Kline R. B. (2005). Principles and Practice of Structural Equation Modeling. New York, NY: The Guilford Pres.

[ref88] Kokandy R. (2021). Teachers’ perceptions of using digital gaming in classrooms. Int. J. Educ. Technol. Learn. 11, 6–13. doi: 10.20448/2003.111.6.13

[ref89] Kong Y. Seo Y. S. Zhai L. (2022). Ict and digital reading achievement: a cross-national comparison using PISA 2018 data. Int. J. Educ. Res. 111, 1–9. doi: 10.1016/j.ijer.2021.101912

[ref90] Kunze T. (2016). Spielerezension: Epistory – Typing Chronicles, mit der Macht der Worte die Elemente entfesseln. Medienimpulse 54, 1–6. doi: 10.21243/MI-03-16-15

[ref91] Kuo H. J. Yeomans M. Ruiz D. Lin C.-C. (2025). Purpose matters: video gaming impacts on addiction symptoms and academic performance of students with disabilities. Entertain. Comput. 52, 1–7. doi: 10.1016/j.entcom.2024.100780

[ref92] Lai C. (2023). *Insights into Autonomy and Technology in Language Teaching*. Available at: https://books.google.com/books?hl=tr&lr=&id=kEkREQAAQBAJ&oi=fnd&pg=PR9&dq=Digital+games+often+require+players+to+navigate+complex+tasks,+solve+problems,+and+process+large+amounts+of+text-based+information,+which+can+contribute+to+reading+persistence+and+skill+development+in+autonomy-supportive+contexts&ots=hmsrbmgDTz&sig=10hbtFzzr3aVXCVlRqHcQu9u3Lc (Accessed February 23, 2025).

[ref93] Lawrence A. M. Sherry M. B. (2021). How feedback from an online video game teaches argument writing for environmental action. J. Lit. Res. 53, 29–52. doi: 10.1177/1086296X20986598

[ref94] Lazarides R. Buchholz J. Rubach C. (2018). Teacher enthusiasm and self-efficacy, student-perceived mastery goal orientation, and student motivation in mathematics classrooms. Teach. Teach. Educ. 69, 1–10. doi: 10.1016/j.tate.2017.08.017

[ref95] Lazarides R. Gaspard H. Dicke A.-L. (2019). Dynamics of classroom motivation: teacher enthusiasm and the development of math interest and teacher support. Learn. Instr. 60, 126–137. doi: 10.1016/j.learninstruc.2018.01.012

[ref96] Lei H. Cui Y. Chiu M. M. (2018). The relationship between teacher support and students’ academic emotions: a meta-analysis. Front. Psychol. 8, 1–12. doi: 10.3389/fpsyg.2017.02288, 29403405 PMC5786576

[ref97] Li X. Chu S. K. W. (2021). Exploring the effects of gamification pedagogy on children’s reading: a mixed-method study on academic performance, reading-related mentality and behaviors, and sustainability. Br. J. Educ. Technol. 52, 160–178. doi: 10.1111/bjet.13057

[ref98] Li L. Hew K. F. Du J. (2024). Gamification enhances student intrinsic motivation, perceptions of autonomy and relatedness, but minimal impact on competency: a meta-analysis and systematic review. Educ. Tech. Res. Dev. 72, 765–796. doi: 10.1007/s11423-023-10337-7

[ref99] Li J. Xue E. Guo S. (2025). The effects of PISA on global basic education reform: a systematic literature review. Humanit. Soc. Sci. Commun. 12:106. doi: 10.1057/s41599-025-04403-z

[ref100] Li X. Yang Y. Chu S. K. W. (2024). How does gamification bring long-term sustainable effects on children’s learning? Implications from a crossover quasi-experimental study. Educ. Tech. Res. Dev. 72, 1357–1381. doi: 10.1007/s11423-023-10341-x

[ref101] Lieury A. Lorant S. Trosseille B. Champault F. Vourc’h R. (2016). Video games vs. reading and school/cognitive performances: a study on 27000 middle school teenagers. Educ. Psychol. 36, 1560–1595. doi: 10.1080/01443410.2014.923556

[ref102] Little T. D. Card N. A. Bovaird J. A. Preacher K. J. Crandall C. S. (2007). “Structural equation modeling of mediation and moderation with contextual factors,” in Modeling Contextual Effects in Longitudinal Studies, eds. Little T. D. Bovaird J. A. Card N. A. (New York: Psychology Press), 207–230.

[ref103] Liu L. Hwang G. (2024). Effects of metalinguistic corrective feedback on novice EFL students’ digital game-based grammar learning performances, perceptions and behavioural patterns. Br. J. Educ. Technol. 55, 687–711. doi: 10.1111/bjet.13400

[ref104] Liu T. Israel M. (2022). Uncovering students’ problem-solving processes in game-based learning environments. Comput. Educ. 182, 1–14. doi: 10.1016/j.compedu.2022.104462

[ref105] Liu C.-C. Lin Y.-Y. Lo F. Chang C.-H. Lin H.-M. (2024). From readers to players: exploring student engagement in a gamified metaverse and its effect on reading interest. Educ. Inf. Technol. 30, 421–447. doi: 10.1007/s10639-024-13068-1

[ref106] Lo N. P.-K. (2024). Cross-cultural comparative analysis of student motivation and autonomy in learning: perspectives from Hong Kong and the United Kingdom. Front. Educ. 9, 1–16. doi: 10.3389/feduc.2024.1393968

[ref107] Lo-Philip S. W. Y. (2024). Digital close reading for lower primary students. Read. Teach. 77, 850–860. doi: 10.1002/trtr.2304

[ref108] Luo S. King R. B. Wang F. Leung S. O. (2024). English digital reading achievement for east Asian students: identifying the key predictors using a machine learning approach. Asia Pac. J. Educ. 2024, 1–17. doi: 10.1080/02188791.2024.2398120

[ref109] Lyu Y. (2024). Gamification as a Bridge Between Intrinsic and Extrinsic Motivation in Chinese post- Secondary Education. Montréal: McGill University.

[ref110] Ma L. Luo H. Xiao L. (2021). Perceived teacher support, self-concept, enjoyment and achievement in reading: a multilevel mediation model based on PISA 2018. Learn. Individ. Differ. 85:101947. doi: 10.1016/j.lindif.2020.101947

[ref111] Ma L. Xiao L. Jiao Y. (2023). Mediation of reading enjoyment between teacher feedback and reading achievement: cross-cultural generalizability across 75 countries/economies. J. Exp. Educ. 92, 431–451. doi: 10.1080/00220973.2023.2208063

[ref112] Mackinnon S. Curtis R. O’Connor R. (2022). Tutorial in longitudinal measurement invariance and cross-lagged panel models using Lavaan. MP 6, 1–20. doi: 10.15626/MP.2020.2595

[ref113] Magpusao J. R. (2024). Gamification and game-based learning in primary education: a bibliometric analysis. Comput. Child. 3:182. doi: 10.29333/cac/14182

[ref114] Manzano León A. Rodríguez Ferrer J. M. Aguilar Parra J. M. Fernández Campoy J. M. Trigueros R. Martínez Martínez A. M. (2022). Play and learn: influence of gamification and game-based learning in the reading processes of secondary school students. Rev. Psicodidact. 27, 38–46. doi: 10.1016/j.psicoe.2021.08.001

[ref115] Marzal M.-Á. Cardama S. M. (2021). Gamification as a strategy for visual literacy skills-based education: a proposal for educational libraries. J. Libr. Inf. Serv. Distance Learn. 15, 236–252. doi: 10.1080/1533290X.2021.2005215

[ref116] Matallaoui A. Hanner N. Zarnekow R. (2017). “Introduction to gamification: foundation and underlying theories,” in Gamification, eds. Stieglitz S. Lattemann C. Robra-Bissantz S. Zarnekow R. Brockmann T. (Cham: Springer International Publishing), 3–18.

[ref117] Montgomery J. L. Baker W. (2007). Teacher-written feedback: student perceptions, teacher self-assessment, and actual teacher performance. J. Second. Lang. Writ. 16, 82–99. doi: 10.1016/j.jslw.2007.04.002

[ref119] Muthén B. Asparouhov T. (2018). Recent methods for the study of measurement invariance with many groups: alignment and random effects. Sociol. Methods Res. 47, 637–664. doi: 10.1177/0049124117701488

[ref120] Ng D. T. K. Xinyu C. Leung J. K. L. Chu S. K. W. (2024). Fostering students’ AI literacy development through educational games: AI knowledge, affective and cognitive engagement. Comput. Assist. Learn. 40, 2049–2064. doi: 10.1111/jcal.13009

[ref121] Nietfeld J. L. (2018). “The role of self-regulated learning in digital games,” in Handbook of Self-Regulation of Learning and Performance, eds. Schunk D. H. Greene J. A. (New York: Routledge, Taylor & Francis Group), 271–284.

[ref122] OECD (2018). PISA 2018 Technical Report. Paris: OECD Publishing.

[ref123] OECD (2019a). PISA 2018 Results (Volume I): What Students Know and Can Do. Paris: OECD.

[ref124] OECD (2019b). PISA 2018 Results (Volume III): What School Life Means for Students’ Lives. Paris: OECD.

[ref125] OECD (2019c). PISA 2018 Results (Volume III): What School Life Means for Students’ Lives. Paris: OECD Publishing.

[ref126] OECD (2021). 21st-Century Readers: Developing Literacy Skills in a Digital World. Paris: OECD Publishing.

[ref127] OECD (2023). PISA 2022 Results (Volume I): The state of Learning and Equity in Education. Paris: OECD Publishing.

[ref128] Ostovar-Namaghi S. A. Morady Moghaddam M. Rad E. (2024). The effect of interactive games on English language learners’ reading comprehension and attitudes. Asia Pac. Educ. Rev. 25, 399–409. doi: 10.1007/s12564-023-09883-9

[ref129] Papadakis S. (2018). The use of computer games in classroom environment. IJTCS 9, 1–25. doi: 10.1504/IJTCS.2018.090191

[ref130] Phillips R. S. (2024). OECD’S programme of international student assessment (PISA) does not include Canada. J. Acad. Perspect. 17, 85–115.

[ref131] Pianta R. C. (2013). “Consistent environmental stimulation from birth to elementary school: the combined contribution of different settings on school achievement,” in Wellbeing: A Complete Reference Guide, ed. Cooper C. L. (Chichester, UK: Wiley-Blackwell), 1–24.

[ref132] Qiao S. Chu S. K. W. Shen X. Yeung S. S. (2022). The impact of an online gamified approach embedded with self-regulated learning support on students’ reading performance and intrinsic motivation: a randomized controlled trial. Comput. Assist. Learn. 38, 1379–1393. doi: 10.1111/jcal.12684

[ref133] Rasmusson M. Åberg-Bengtsson L. (2015). Does performance in digital reading relate to computer game playing? A study of factor structure and gender patterns in 15-year-olds’ reading literacy performance. Scand. J. Educ. Res. 59, 691–709. doi: 10.1080/00313831.2014.965795

[ref134] Reeve J. (2012). “A self-determination theory perspective on student engagement,” in Handbook of Research on Student Engagement, eds. Christenson S. L. Reschly A. L. Wylie C. (Boston, MA: Springer), 149–172.

[ref135] Roback P. Legler J. (2021). Beyond Multiple Linear Regression: Applied Generalized linear Models and Multilevel Models in R. 1st Edn. Boca Raton, FL, USA: Chapman and Hall/CRC.

[ref136] Robitzsch A. Lüdtke O. (2023). Why full, partial, or approximate measurement invariance are not a prerequisite for meaningful and valid group comparisons. Struct. Equ. Modeling 30, 859–870. doi: 10.1080/10705511.2023.2191292

[ref137] Ronimus M. Eklund K. Pesu L. Lyytinen H. (2019). Supporting struggling readers with digital game-based learning. Educ. Technol. Res. Dev. 67, 639–663. doi: 10.1007/s11423-019-09658-3

[ref138] Rosenman R. Tennekoon V. Hill L. G. (2011). Measuring bias in self-reported data. Int. J. Behav. Healthcare Res. 2, 320–332. doi: 10.1504/IJBHR.2011.043414, 25383095 PMC4224297

[ref139] Rosseel Y. (2012). Lavaan: an R package for structural equation modeling. J. Stat. Soft. 48, 1–36. doi: 10.18637/jss.v048.i02

[ref140] Rubin D. B. (2018). “Multiple imputation,” in Flexible Imputation of Missing Data, ed. Van Buuren S.. 2nd ed (Boca Raton: CRC Press), 29–62.

[ref141] Ryan R. M. Deci E. L. (2017). Self-Determination Theory: Basic Psychological Needs in Motivation, Development and Wellness. New York: The Guilford Press.

[ref142] Ryan R. M. Deci E. L. (2020). Intrinsic and extrinsic motivation from a self-determination theory perspective: definitions, theory, practices, and future directions. Contemp. Educ. Psychol. 61, 1–11. doi: 10.1016/j.cedpsych.2020.101860

[ref143] Saglam M. H. Goktenturk T. (2024). Mathematically high and low performances tell us different stories: uncovering motivation-related factors via the ecological model. Learn. Individ. Differ. 114, 1–12. doi: 10.1016/j.lindif.2024.102513

[ref144] Saglam M. H. Göktentürk T. Demir I. Yazıcı E. (2023). Environmental factors for the advancement of teachers’ self-efficacy in professional development. J. Intelligence 11, 1–14. doi: 10.3390/jintelligence11020028, 36826926 PMC9962100

[ref145] Sağlam M. H. Göktentürk T. Lo C. O. (2023). The factors explaining reading success of academically gifted readers through the ecological model. Gift. Educ. Int. 40, –66. doi: 10.1177/02614294231170265

[ref146] Sánchez-Mena A. Martí-Parreño J. (2017). Teachers’ acceptance of educational video games: a comprehensive literature review. J. E-Learn. Knowl. Soc. 13, 1–17. doi: 10.20368/1971-8829/139

[ref147] Schleicher A. (2023). PISA 2022 Insights and Interpretations. Paris: OECD.

[ref148] Schrader C. Bastiaens T. J. (2012). The influence of virtual presence: effects on experienced cognitive load and learning outcomes in educational computer games. Comput. Hum. Behav. 28, 648–658. doi: 10.1016/j.chb.2011.11.011

[ref149] Schulz Van Endert T. (2021). Addictive use of digital devices in young children: associations with delay discounting, self-control and academic performance. PLoS One 16, e0253058–e0253012. doi: 10.1371/journal.pone.0253058, 34157026 PMC8219150

[ref150] Schwartz S. H. (2009). “Culture matters: National value cultures, sources, and consequences,” in Understanding Culture: Theory, Research, and Application, eds. Wyer R. S. Chiu C. Hong Y. (New York: Psychology Press), 127–150.

[ref151] Sivakumar A. (2024). “Gamification for teaching beyond for creative learners,” in Transformative Digital Technology for Disruptive Teaching and Learning, eds. Kaliraj P. Singaravelu G. Devi T. (New York: CRC Press), 101–112.

[ref152] Sofiana N. Mubarok H. (2020). The impact of Englishgame-based mobile application on students’ reading achievement and learning motivation. Int. J. Instr. 13, 247–258. doi: 10.29333/iji.2020.13317a

[ref153] Suárez-Mesa A. M. Gómez R. L. (2024). Does teachers’ motivation have an impact on students’ scientific literacy and motivation? An empirical study in Colombia with data from PISA 2015. Large Scale Assess. Educ. 12:1. doi: 10.1186/s40536-023-00190-8

[ref154] Sun C.-T. Chou K.-T. Yu H. C. (2022). Relationship between digital game experience and problem-solving performance according to a PISA framework. Comput. Educ. 186:104534. doi: 10.1016/j.compedu.2022.104534

[ref155] Tanouri A. Kennedy A.-M. Veer E. (2024). An implementation framework for transformative gamification services. Behav. Inf. Technol. 43, 2118–2150. doi: 10.1080/0144929X.2023.2241560

[ref156] Tao Y. Meng Y. Gao Z. Yang X. (2022). Perceived teacher support, student engagement, and academic achievement: a meta-analysis. Educ. Psychol. 42, 401–420. doi: 10.1080/01443410.2022.2033168

[ref157] Tay J. Goh Y. M. Safiena S. Bound H. (2022). Designing digital game-based learning for professional upskilling: a systematic literature review. Comput. Educ. 184, 1–15. doi: 10.1016/j.compedu.2022.104518

[ref158] Tibbe T. D. Montoya A. K. (2022). Correcting the Bias correction for the bootstrap confidence interval in mediation analysis. Front. Psychol. 13, 1–21. doi: 10.3389/fpsyg.2022.810258, 35712166 PMC9197131

[ref159] Toh W. Kirschner D. (2023). Developing social-emotional concepts for learning with video games. Comput. Educ. 194, 1–16. doi: 10.1016/j.compedu.2022.104708

[ref160] Troy J. (1994). Reader Rabbit 3. Booklist 90, 1277–1278.

[ref161] UNESCO (2022). International Standard Classification of Teacher Training Programmes (ISCED-T 2021). Montreal, QC, Canada: UNESCO.

[ref162] Urdan T. Bruchmann K. (2018). Examining the academic motivation of a diverse student population: a consideration of methodology. Educ. Psychol. 53, 114–130. doi: 10.1080/00461520.2018.1440234

[ref163] Usta Ç. Şahan A. (2024). Beyond the classroom: integrating video games as extracurricular English activities. Int. J. Educ. Spectr. 6, 181–190. doi: 10.47806/ijesacademic.1407158

[ref164] Van Den Bergh L. Ros A. Beijaard D. (2013). Teacher feedback during active learning: current practices in primary schools. Brit. J. of Edu. Psychol. 83, 341–362. doi: 10.1111/j.2044-8279.2012.02073.x, 23692539

[ref165] Van Der Stede W. A. (2014). A manipulationist view of causality in cross-sectional survey research. Account. Organ. Soc. 39, 567–574. doi: 10.1016/j.aos.2013.12.001

[ref166] Van Dijk W. Schatschneider C. Al Otaiba S. Hart S. A. (2022). Assessing measurement invariance across multiple groups: when is fit good enough? Educ. Psychol. Meas. 82, 482–505. doi: 10.1177/00131644211023567, 35444334 PMC9014728

[ref167] Vanbecelaere S. Van Den Berghe K. Cornillie F. Sasanguie D. Reynvoet B. Depaepe F. (2020). The effects of two digital educational games on cognitive and non-cognitive math and reading outcomes. Comput. Educ. 143, 1–15. doi: 10.1016/j.compedu.2019.103680

[ref168] Villalta M. Gajardo I. Nussbaum M. Andreu J. J. Echeverría A. Plass J. L. (2011). Design guidelines for classroom multiplayer Presential games (CMPG). Comput. Educ. 57, 2039–2053. doi: 10.1016/j.compedu.2011.05.003

[ref169] Villegas-Reimers E. (2003). Teacher Professional Development: an International Review of the Literature. Paris: International Institute for Educational Planning.

[ref171] Weinstein A. M. (2010). Computer and video game addiction—a comparison between game users and non-game users. Am. J. Drug Alcohol Abuse 36, 268–276. doi: 10.3109/00952990.2010.491879, 20545602

[ref172] Wentzel K. Skinner E. (2022). The other half of the story: the role of social relationships and social contexts in the development of academic motivation. Educ. Psychol. Rev. 34, 1865–1876. doi: 10.1007/s10648-022-09713-1

[ref173] Wood J. (2021). A dialogic technology-mediated model of feedback uptake and literacy. Assess. Eval. High. Educ. 46, 1173–1190. doi: 10.1080/02602938.2020.1852174

[ref174] Wu L. Valcke M. Van Keer H. (2021). Supporting struggling readers at secondary school: an intervention of reading strategy instruction. Read. Writ. 34, 2175–2201. doi: 10.1007/s11145-021-10144-7

[ref175] Xia Y. Yang Y. (2019). RMSEA, CFI, and TLI in structural equation modeling with ordered categorical data: the story they tell depends on the estimation methods. Behav. Res. Methods 51, 409–428. doi: 10.3758/s13428-018-1055-2, 29869222

[ref176] Yang L. Chiu M. M. Yan Z. (2021). The power of teacher feedback in affecting student learning and achievement: insights from students’ perspective. Educ. Psychol. 41, 821–824. doi: 10.1080/01443410.2021.1964855

[ref177] Yang Y.-F. Tseng C. C. Lai S.-C. (2024). Collaborative digital game design and play to facilitate college students’ creativity development. Interact. Learn. Environ. 2024, 1–20. doi: 10.1080/10494820.2024.2368875

[ref178] Yilmaz V. Tunca B. (2024). Structural model proposal to explain online game addiction. Entertain. Comput. 48, 1–13. doi: 10.1016/j.entcom.2023.100611

[ref179] Yu Z. Gao M. Wang L. (2021). The effect of educational games on learning outcomes, student motivation, engagement and satisfaction. J. Educ. Comput. Res. 59, 522–546. doi: 10.1177/0735633120969214

[ref180] Zhang W. Gu L. (2022). The role of computer game playing and reading attitudes in digital reading achievement: evidence from Hong Kong 15-year-olds. RPTEL 18, 1–27. doi: 10.58459/rptel.2023.18019

[ref181] Zhang H. Li F. Yan H. (2024). Causal mechanisms of the psychological needs for online gamified learning and the impact on learning engagement among college students. Interact. Learn. Environ. 33, 2597–2618. doi: 10.1080/10494820.2024.2412088

[ref182] Zhang S. Xu J. Chen H. Jiang L. Yi X. (2024). Influence of teacher autonomy support in feedback on high school students’ feedback literacy: the multiple mediating effects of basic psychological needs and intrinsic motivation. Front. Psychol. 15, 1–9. doi: 10.3389/fpsyg.2024.1411082, 39193032 PMC11347947

[ref183] Zhao X. Lynch J. G. Chen Q. (2010). Reconsidering baron and Kenny: myths and truths about mediation analysis. J. Consum. Res. 37, 197–206. doi: 10.1086/651257

[ref184] Zou H. Deng Y. Wang H. Yu C. Zhang W. (2022). Perceptions of school climate and internet gaming addiction among Chinese adolescents: the mediating effect of deviant peer affiliation. IJERPH 19, 1–8. doi: 10.3390/ijerph19063604, 35329291 PMC8954293

