# Supplementary material for: Mediating effects of student-perceived instructional practices in the digital gaming—reading performance relationship: a multilevel analysis of PISA 2018
Source: Front Psychol. 2026 Apr 10;17:1766681. doi: 10.3389/fpsyg.2026.1766681 (PMC13106611; doi:10.3389/fpsyg.2026.1766681)
Supplement: Supplementary file 1 [file Data_sheet_1.pdf]

Supplementary Material

Content of Tables

Table 1. Descriptive statistics of variables 2
Table 2. Response types, missing values, and Cronbach's Alpha coefficients 3
Table 3. Descriptive statistics of countries 5
Table 4. Scale reliabilities of the factors for each country 7
Table 5. Sample syntax for multilevel SEM and measurement invariance 9
Table 6. Sample syntax for missing-data sensitivity analysis 10
Table 7. Measurement invariance analysis results for configural, metric, and scalar models 11
Table 8. Gender-adjusted robustness check results for direct and indirect paths 12

Content of Figures

Figure 1. Scatterplots for linearity 13
Figure 2. Q-Q-plots for normality 14

Table 1. Descriptive statistics of variables

Category	Variables	Mean	Std. Deviation	Range
Student level Independent variable	*Factor*. Playing digital games	2.50	.89	1-5
	Item. Use digital devices outside of school: Playing one-player games.	2.74	1.08	
	Item. Use digital devices outside of school: Playing collaborative online games.	2.64	1.15	
	Item. Use digital devices outside of school: Playing online games via Social Networks (e.g. <Farmville>, <The Sims Social>).	2.12	1.09	
School level Mediating variables	*Factor*. Teacher feedback	2.38	.81	1-4
	Item. How often during <test language lessons>: The teacher gives me feedback on my strengths in this subject.	2.21	.92	
	Item. How often during <test language lessons>: The teacher tells me in which areas I can still improve.	2.41	.92	
	Item. How often during <test language lessons>: The teacher tells me how I can improve my performance.	2.51	.93	
	Factor. Teacher stimulation for reading engagement	2.65	.76	1-4
	Item. In your <test language lessons>, how often: The teacher encourages students to express their opinion about a text.	2.75	.91	
	Item. In your <test language lessons>, how often: The teacher helps students relate the stories they read to their lives.	2.44	.95	
	Item. In your <test language lessons>, how often: The teacher shows students how the information in texts builds on [...]	2.66	.88	
	Item. In your <test language lessons>, how often: The teacher poses questions that motivate students to participate actively.	2.77	.93	
	Factor. Teacher support (Reverse Coded)	3.18	.76	1-4
	Item. How often during <test language lessons>: The teacher shows an interest in every student's learning. (Reverse Coded)	3.14	.91	
	Item. How often during <test language lessons>: The teacher gives extra help when students need it. (Reverse Coded)	3.18	.89	
	Item. How often during <test language lessons>: The teacher helps students with their learning. (Reverse Coded)	3.26	.87	
	Item. How often during <test language lessons>: The teacher continues teaching until the students understand. (Reverse Coded)	3.14	.92	

Table 2. Response types, missing values, and Cronbach's Alpha coefficients

Factors	Codes	Items	Response Type	Missing Value Ratio	Cronbach's Alpha
**Digital Gaming**	IC008Q01TA	Use digital devices outside of school: Playing one-player games.	Five-point scale: (1) never or hardly ever (2) once or twice a month (3) once or twice a week (4) almost every day (5) every day	44.9%	.73
	IC008Q02TA	Use digital devices outside of school: Playing collaborative online games.		45.3%	
	IC008Q07NA	Use digital devices outside of school: Playing online games via Social Networks (e.g. <Farmville>, <The Sims Social>).		45.3%	
**Teacher Feedback (TF-Academic)**	ST104Q02NA	How often during <test language lessons>: The teacher gives me feedback on my strengths in this subject.	4-point scale: (1) never or almost never (2) some lessons (3) many lessons (4) Every lesson or almost every lesson.	9.8%	.85
	ST104Q03NA	How often during <test language lessons>: The teacher tells me in which areas I can still improve.		9.8%	
	ST104Q04NA	How often during <test language lessons>: The teacher tells me how I can improve my performance.		10%	
**Teachers' Stimulation of Reading Engagement (TSRE-Academic)**	ST152Q05IA	In your <test language lessons>, how often: The teacher encourages students to express their opinion about a text.	4-point scale: (1) never or hardly ever (2) in some lessons (3) in most lessons (4) in all lessons.	6.7%	.85
	ST152Q06IA	In your <test language lessons>, how often: The teacher helps students relate the stories they read to their lives.		7.2%	
	ST152Q07IA	In your <test language lessons>, how often: The teacher shows students how the information in texts builds on [...]		7.4%	
	ST152Q08IA	In your <test language lessons>, how often: The teacher poses questions that motivate students to participate actively.		7.3%	

Table 2. (*continued*)

**Teacher Support (TS-Academic)**	ST100Q01TA	How often during <test language lessons>: The teacher shows an interest in every student's learning. (Reverse Coded)	4-point scale: (1) every lesson (2) most lessons (3) some lessons (4) never or hardly ever.	8.6%	.87
	ST100Q02TA	How often during <test language lessons>: The teacher gives extra help when students need it. (Reverse Coded)		8.9%	
	ST100Q03TA	How often during <test language lessons>: The teacher helps students with their learning. (Reverse Coded)		9%	
	ST100Q04TA	How often during <test language lessons>: The teacher continues teaching until the students understands. (Reverse Coded)		8.9%	

Table 3. Descriptive statistics of countries

Country	N (schools)	N (students)	Female %	Male %
Albania	327	6359	49.8	50.2
Argentina	455	11975	52.0	48.0
Australia	763	14273	49.6	50.4
Austria	291	6802	48.8	51.2
B-S-J-Z (China)	361	12058	47.9	52.1
Baku (Azerbaijan)	197	6827	47.8	52.2
Belarus	234	5803	47.8	52.2
Belgium	288	8475	50.4	49.6
Bosnia and Herzegovina	213	6480	48.6	51.4
Brazil	597	10691	51.2	48.8
Brunei Darussalam	55	6828	49.5	50.5
Bulgaria	197	5294	47.8	52.2
Canada	821	22651	49.9	50.1
Chile	254	7621	50.0	50.0
Chinese Taipei	192	7243	50.0	50.0
Colombia	247	7522	51.3	48.7
Costa Rica	205	7221	50.1	49.9
Croatia	203	6609	50.1	49.9
Czech Republic	333	7019	50.1	499
Denmark	348	7657	49.8	50.2
Dominican Republic	235	5674	50.9	49.1
Estonia	230	5316	49.9	50.1
Finland	214	5649	49.1	50.9
France	254	6308	48.8	51.2
Georgia	321	5572	48.1	51.9
Germany	223	5451	46.3	53.7
Greece	242	6403	49.6	50.4
Hong Kong	152	6037	48.9	51.1
Hungary	238	5132	50.8	49.2
Iceland	142	3296	50.2	49.8
Indonesia	397	12098	51.6	48.4
Ireland	157	5577	49.8	50.2
Israel	174	6623	53.5	46.5
Italy	542	11785	48.2	51.8
Japan	185	6109	51.1	48.9
Jordan	313	8963	51.5	48.5
Kazakhstan	616	19507	49.1	50.9
Korea	188	6650	48.0	52.0
Kosovo	211	5058	48.6	51.4
Latvia	308	5303	50.6	49.4
Lebanon	313	5614	54.8	45.2
Lithuania	362	6885	49.0	51.0
Luxembourg	44	5230	49.6	50.4

Table 3. (*continued*)

Macao	45	3775	49.3	50.7
Malaysia	191	6111	51.2	48.8
Malta	50	3363	47.9	52.1
Mexico	286	7299	52.4	47.6
Moldova	236	5367	48.8	51.2
Montenegro	61	6666	48.6	51.4
Morocco	179	6814	47.9	52.1
Moscow Region (RUS)	61	2016	48.1	51.9
Netherlands	156	4765	48.9	51.1
New Zealand	192	6173	51.1	48.9
North Macedonia	117	5569	46.6	53.8
Norway	251	5813	49.5	50.5
Panama	253	6270	50.6	49.4
Peru	340	6086	49.3	50.7
Philippines	187	7233	53.5	46.5
Poland	240	5625	50.8	49.2
Portugal	276	5932	49.6	50.4
Qatar	188	13828	50.3	49.7
Romania	170	5075	48.2	51.8
Russian Federation	263	7608	50.7	49.3
Saudi Arabia	234	6136	48.8	51.2
Serbia	187	6609	49.5	50.5
Singapore	166	6676	49.1	50.9
Slovak Republic	376	5965	50.3	49.7
Slovenia	345	6401	46.8	53.2
Spain	1089	35943	50.0	50.0
Sweden	223	5504	50.2	49.8
Switzerland	228	5822	47.9	52.1
Tatarstan (RUS)	239	5816	50.0	50.0
Thailand	290	8633	54.4	45.6
Turkey	186	6890	49.3	50.7
Ukraine	250	5998	47.6	52.4
United Arab Emirates	755	19277	48.7	51.3
United Kingdom	471	13818	50.6	49.4
United States	164	4838	49.1	50.9
Uruguay	189	5263	51.9	48.1

Table 4. Scale reliabilities of the factors for each country

Countries	PDG	TF	TSRE	TS
Albania	.83	.86	.87	.67
Argentina	.75	.87	.85	.64
Australia	.78	.82	.85	.67
Austria	.72	.86	.85	.68
B-S-J-Z (China)	.71	.81	.78	.66
Baku (Azerbaijan)	.74	.79	.79	.63
Belarus	.73	.84	.81	.69
Belgium	.78	.86	.85	.68
Bosnia and Herzegovina	.80	.82	.84	.71
Brazil	.73	.84	.81	.69
Brunei Darussalam	.66	.89	.82	.69
Bulgaria	.76	.84	.81	.69
Canada	.74	.86	.84	.70
Chile	.66	.83	.84	.74
Chinese Taipei	.74	.85	.84	.73
Colombia	.75	.84	.88	.71
Costa Rica	.75	.88	.90	.68
Croatia	.61	.82	.81	.73
Czech Republic	.74	.85	.83	.70
Denmark	.78	.81	.84	.67
Dominican Republic	.71	.87	.87	.73
Estonia	.60	.90	.91	.69
Finland	.76	.88	.90	.72
France	.75	.82	.85	.71
Georgia	.76	.87	.90	.72
Germany	.73	.76	.78	.61
Greece	.79	.83	.87	.66
Hong Kong	.75	.86	.82	.69
Hungary	.72	.83	.79	.66
Iceland	.61	.75	.87	.60
Indonesia	.70	.81	.79	.69
Ireland	.77	.78	.79	.72
Israel	.62	.74	.86	.60
Italy	.74	.76	.85	.61
Japan	.77	.78	.75	.68
Jordan	.73	.81	.78	.67
Kazakhstan	.69	.80	.78	.62
Korea	.72	.84	.76	.72
Kosovo	.71	.82	.82	.68
Latvia	.74	.76	.83	.60
Lebanon	.66	.82	.79	.63
Lithuania	.70	.79	.81	.71
Luxembourg	.67	.76	.78	.66

Table 4. (*continued*)

Macao	.73	.89	.88	.60
Malaysia	.68	.77	.79	.72
Malta	.72	.82	.81	.64
Mexico	.72	.73	.76	.70
Moldova	.76	.80	.80	.64
Montenegro	.61	.84	.78	.63
Morocco	.63	.74	.82	.69
Moscow Region (RUS)	.75	.80	.84	.66
Netherlands	.72	.79	.78	.67
New Zealand	.73	.80	.82	.61
North Macedonia	.68	.73	.78	.69
Norway	.66	.80	.83	.64
Panama	.75	.83	.82	.63
Peru	.78	.82	.81	.69
Philippines	.74	.82	.84	.64
Poland	.73	.81	.79	.70
Portugal	.73	.84	.81	.68
Qatar	.74	.79	.77	.63
Romania	.69	.77	.80	.67
Russian Federation	.72	.81	.83	.68
Saudi Arabia	.77	.77	.79	.68
Serbia	.72	.80	.81	.67
Singapore	.68	.80	.81	.65
Slovak Republic	.76	.80	.81	.66
Slovenia	.74	.84	.83	.69
Spain	.68	.84	.85	.71
Sweden	.76	.83	.84	.72
Switzerland	.73	.87	.86	.66
Tatarstan (RUS)	.71	.83	.82	.70
Thailand	.72	.82	.84	.72
Turkey	.72	.86	.86	.77
Ukraine	.72	.83	.86	.75
United Arab Emirates	.66	.87	.86	.63
United Kingdom	.77	.87	.86	.64
United States	.73	.84	.85	.76
Uruguay	.78	.86	.87	.74

Table 5. Sample syntax for multilevel SEM and measurement invariance

**The Sample Syntax of Multilevel SEM***	**Sample Syntax of Measurement Invariance****
# Define the SEM model for multilevel analysis<br>model <- '<br># Level 1: Within (Student Level)<br>PDG =~ IC008Q01TA + IC008Q02TA + IC008Q07NA<br># Level 2: Between (School Level Student-Perceived Instructional Practices Mediators)<br>TF =~ ST104Q02NA + ST104Q03NA + ST104Q04NA<br>TSRE =~ ST152Q05IA + ST152Q06IA + ST152Q07IA + ST152Q08IA<br>TS =~ ST100Q01TA + ST100Q02TA + ST100Q03TA + ST100Q04TA<br># Covariances between Level 2 mediators<br>TF ~~ TSRE + TS<br>TSRE ~~ TS<br># Regressions<br>PV_READ ~ c * PDG<br># Between-level mediation paths<br>TF ~ a8 * PDG<br>TSRE ~ a9 * PDG<br>TS ~ a10 * PDG<br># Structural Model for Level 2<br>PV_READ ~ b8 * TF + b9 * TSRE + b10 * TS'<br># Fit the two-level model to your data, specifying the cluster and sampling weight variables<br>fit <- sem(model, data = data, cluster = "CLUSTER_ID", sampling.weights = "W_FSTUWT_norm")<br># Summarize the results<br>summary(fit, standardized = TRUE)	model <- '<br># Factor loadings<br>TF =~ ST104Q02NA + ST104Q03NA + ST104Q04NA<br>TSRE =~ ST152Q05IA + ST152Q06IA + ST152Q07IA + ST152Q08IA<br>TS =~ ST100Q01TA + ST100Q02TA + ST100Q03TA + ST100Q04TA<br>'<br><br># Step 4: Specify the configural invariance model<br>configural_model <- '<br>TF =~ ST104Q02NA + ST104Q03NA + ST104Q04NA<br>TSRE =~ ST152Q05IA + ST152Q06IA + ST152Q07IA + ST152Q08IA<br>TS =~ ST100Q01TA + ST100Q02TA + ST100Q03TA + ST100Q04TA<br>'<br># Step 5: Test the model<br>configural_fit <- cfa(configural_model, data = data, group = "CNT")<br># Summarize the model fit<br>summary(configural_fit, fit.measures = TRUE)

[*] The code shown here is illustrative sample syntax for the final two-level analysis strategy. The model was estimated separately for PV1READ through PV10READ. In each run, a school-level cluster identifier based on CNTSCHID (combined with country where needed) was specified as the clustering variable, and normalized W_FSTUWT_norm was specified as the sampling-weight variable. Final reported estimates were pooled across the ten plausible value runs according to Rubin's rules.

[**] Measurement invariance was tested separately for each classification system (CNT, WCR, and TTS) using sequential configural, metric, and scalar multi-group CFA models. Final results supported configural, metric, and scalar invariance across all three classification systems.

Table 6. Sample syntax for missing-data sensitivity analysis

Listwise deletion	Sample Syntax of Measurement Invariance
# Create a dataset with complete cases only for digital gaming and student-perceived instructional practices<br>MultipleLDA <-<br>MultipleLD[complete.cases(MultipleLD[, c(<br>"IC008Q01TA", "IC008Q02TA", "IC008Q07NA",<br>"ST104Q02NA", "ST104Q03NA", "ST104Q04NA",<br>"ST152Q05IA", "ST152Q06IA", "ST152Q07IA",<br>"ST152Q08IA",<br>"ST100Q01TA", "ST100Q02TA", "ST100Q03TA",<br>"ST100Q04TA")]),]<br># Check how many cases were removed<br>nrow(MultipleLD) # Original dataset size<br># [1] 612004<br>nrow(MultipleLDA) # After listwise deletion<br># [1] 314224<br># Check the percentage of cases removed<br>percent_removed <- (1 - (nrow(MultipleLDA) / nrow(MultipleLD))) * 100<br>print(paste("Percentage of cases removed due to listwise deletion:", round(percent_removed, 2), "%"))<br># [1] "Percentage of cases removed due to listwise deletion: 48.66 %"<br># Compare means and standard deviations before and after deletion<br>summary(MultipleLD[, c(<br>"IC008Q01TA", "IC008Q02TA", "IC008Q07NA",<br>"ST104Q02NA", "ST104Q03NA", "ST104Q04NA",<br>"ST152Q05IA", "ST152Q06IA", "ST152Q07IA",<br>"ST152Q08IA",<br>"ST100Q01TA", "ST100Q02TA", "ST100Q03TA",<br>"ST100Q04TA")])	# Define the imputation methods for all variables<br>method <- rep("", ncol(MultipleLD)) # Default: no imputation<br>names(method) <- colnames(MultipleLD)<br># Use predictive mean matching (pmm) for MNAR variables to reduce extreme values<br>method[mnar_vars] <- "pmm"<br># Run sensitivity analysis using the pattern-mixture model with `pmm`<br>set.seed(123)<br>mnar_model <- mice(MultipleLD,<br>method = method,<br>predictorMatrix = predMatrix,<br>delta = 0.2, # Sensitivity parameter adjustment<br>maxit = 5, # Number of iterations<br>m = 5) # Number of imputed datasets<br># Extract the MNAR-imputed dataset<br>MultipleMNAR <- complete(mnar_model)<br># Check summary statistics of imputed values<br>summary(MultipleMNAR[, mnar_vars])

Table 7. Measurement invariance analysis results for configural, metric, and scalar models

Classification system	Invariance level	χ^2	df	CFI	TLI	RMSEA	SRMR
CNT	Configural	82148.054	3239	.977	.969	.056	.029
CNT	Metric	114706.162	3863	.967	.963	.061	.039
CNT	Scalar	255085.956	4487	.926	.928	.075	.056
WCR	Configural	32161.000	328	.988	.984	.040	.022
WCR	Metric	36683.000	384	.987	.985	.039	.026
WCR	Scalar	77702.000	440	.973	.973	.052	.033
TTS	Configural	26387.000	82	.990	.987	.036	.019
TTS	Metric	27699.000	90	.990	.988	.035	.020
TTS	Scalar	36745.000	98	.987	.985	.038	.022

Table 8. Gender-adjusted robustness check results for direct and indirect paths

Path	Main model B [95% CI]	Main β	Gender-adjusted model B [95% CI]	Gender-adjusted β
**Direct model**				
PDG -> READ (between)	-.731 [-.794, -.668]	-.214	-.730 [-.793, -.667]	-.214
PDG -> READ (within)	-.088 [-.091, -.085]	-.090	-.088 [-.091, -.085]	-.090
**Mediation model direct paths**				
PDG -> TF	.409 [.373, .446]	.242	.410 [.374, .446]	.242
PDG -> TSRE	.235 [.200, .270]	.139	.237 [.202, .272]	.140
PDG -> TS	-.479 [-.541, -.418]	-.152	-.481 [-.542, -.419]	-.153
TF -> READ	-.249 [-.320, -.178]	-.123	-.199 [-.269, -.128]	-.098
TSRE -> READ	.405 [.338, .473]	.201	.326 [.258, .394]	.162
TS -> READ	.149 [.116, .182]	.137	.139 [.106, .172]	.128
PDG -> READ (between)	-.650 [-.715, -.584]	-.190	-.658 [-.724, -.592]	-.193
PDG -> READ (within)	-.088 [-.091, -.085]	-.090	-.088 [-.091, -.085]	-.090
**Mediation model indirect paths**				
PDG -> TF -> READ	-.102 [-.132, -.072]	-	-.082 [-.111, -.052]	-
PDG -> TSRE -> READ	.095 [.074, .117]	-	.077 [.057, .097]	-
PDG -> TS -> READ	-.071 [-.089, -.053]	-	-.067 [-.085, -.049]	-

Note. Main model = final model reported in the manuscript. Gender-adjusted model = robustness model including gender at the student level and school gender composition at the between-school level. B = pooled unstandardized estimate across plausible-value runs with Rubin-style pooling; β = pooled standardized coefficient for direct paths. Indirect effects are reported as pooled unstandardized estimates.

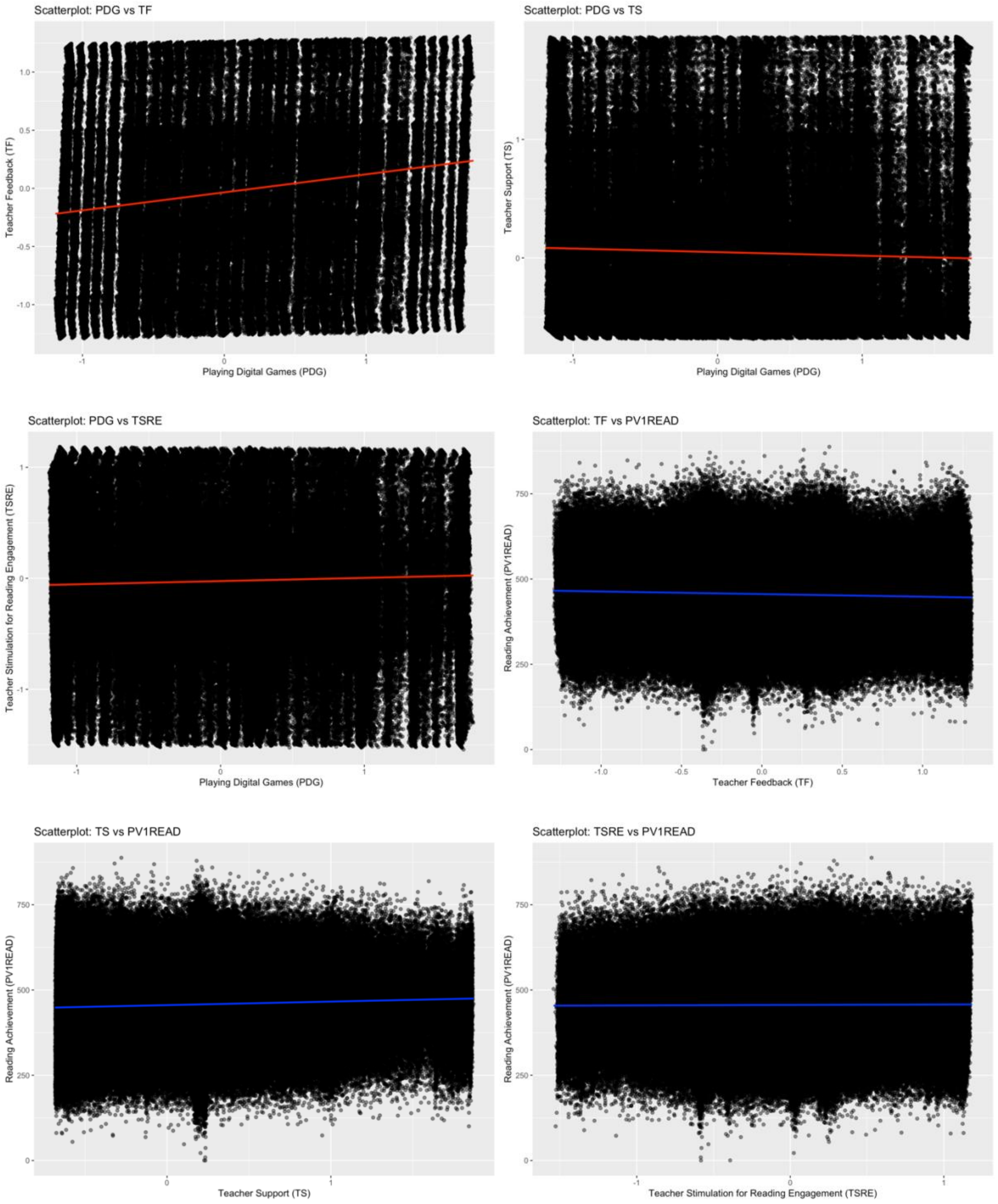

Figure 1. Scatterplots for linearity

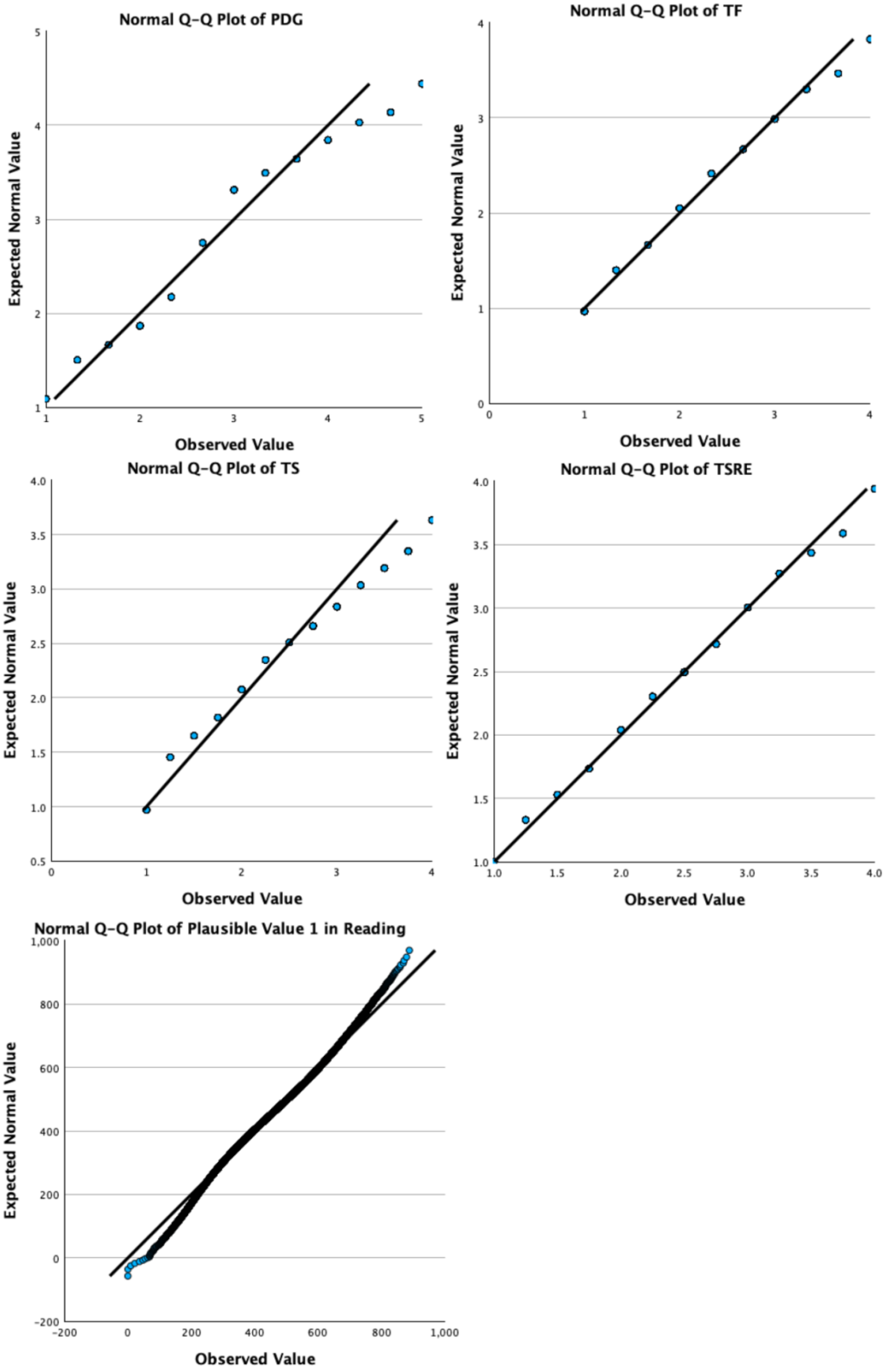

Figure 2. Q-Q-plots for normality
